# Multi-modal molecular determinants of clinically relevant osteoporosis subtypes

**DOI:** 10.1038/s41421-024-00652-5

**Published:** 2024-03-12

**Authors:** Chunchun Yuan, Xiang-Tian Yu, Jing Wang, Bing Shu, Xiao-Yun Wang, Chen Huang, Xia Lv, Qian-Qian Peng, Wen-Hao Qi, Jing Zhang, Yan Zheng, Si-Jia Wang, Qian-Qian Liang, Qi Shi, Ting Li, He Huang, Zhen-Dong Mei, Hai-Tao Zhang, Hong-Bin Xu, Jiarui Cui, Hongyu Wang, Hong Zhang, Bin-Hao Shi, Pan Sun, Hui Zhang, Zhao-Long Ma, Yuan Feng, Luonan Chen, Tao Zeng, De-Zhi Tang, Yong-Jun Wang

**Affiliations:** 1 Longhua Hospital, Shanghai University of Traditional Chinese Medicine, Shanghai, China; 2 Key Laboratory of Theory and Therapy of Muscles and Bones, Ministry of Education, Shanghai, China; 3 Spine Institute, Shanghai Academy of Traditional Chinese Medicine, Shanghai, China; 4 Clinical Research Center, Shanghai Sixth People’s Hospital Affiliated to Shanghai Jiao Tong University School of Medicine, Shanghai, China; 5 Shanghai Geriatric Institute of Chinese Medicine, Shanghai, China; 6 Shanghai University of Traditional Chinese Medicine, Shanghai, China; 7 Shanghai Research Institute of Acupuncture and Meridian, Shanghai, China; 8 Hudong Hospital of Shanghai, Shanghai, China; 9 CAS Key Laboratory of Computational Biology, Shanghai Institute of Nutrition and Health, University of Chinese Academy of Sciences, Chinese Academy of Sciences, Shanghai, China; 10 Ministry of Education Key Laboratory of Contemporary Anthropology, Department of Anthropology and Human Genetics, School of Life Science, Fudan University, Shanghai, China; 11 State Key Laboratory of Genetic Engineering, School of Life Sciences and Human Phenome Institute, Fudan University, Shanghai, China; 12 Green Valley (Shanghai) Pharmaceuticals Co., Ltd., Shanghai, China; 13 State Key Laboratory of Cell Biology, Shanghai Institute of Biochemistry and Cell Biology, Center for Excellence in Molecular Cell Science, Chinese Academy of Sciences, University of Chinese Academy of Sciences, Shanghai, China; 14 Guangzhou National Laboratory, Guangzhou, China

**Keywords:** Mechanisms of disease, Bioinformatics

## Abstract

Due to a rapidly aging global population, osteoporosis and the associated risk of bone fractures have become a wide-spread public health problem. However, osteoporosis is very heterogeneous, and the existing standard diagnostic measure is not sufficient to accurately identify all patients at risk of osteoporotic fractures and to guide therapy. Here, we constructed the first prospective multi-omics atlas of the largest osteoporosis cohort to date (longitudinal data from 366 participants at three time points), and also implemented an explainable data-intensive analysis framework (DLSF: Deep Latent Space Fusion) for an omnigenic model based on a multi-modal approach that can capture the multi-modal molecular signatures (M3S) as explicit functional representations of hidden genotypes. Accordingly, through DLSF, we identified two subtypes of the osteoporosis population in Chinese individuals with corresponding molecular phenotypes, i.e., clinical intervention relevant subtypes (CISs), in which bone mineral density benefits response to calcium supplements in 2-year follow-up samples. Many snpGenes associated with these molecular phenotypes reveal diverse candidate biological mechanisms underlying osteoporosis, with xQTL preferences of osteoporosis and its subtypes indicating an omnigenic effect on different biological domains. Finally, these two subtypes were found to have different relevance to prior fracture and different fracture risk according to 4-year follow-up data. Thus, in clinical application, M3S could help us further develop improved diagnostic and treatment strategies for osteoporosis and identify a new composite index for fracture prediction, which were remarkably validated in an independent cohort (166 participants).

## Introduction

Osteoporosis is conventionally regarded as a systemic skeletal disease accompanied by low bone mass, microarchitectural deterioration of bone tissue, bone fragility and susceptibility to fracture^1^. Osteoporosis and its increased risk for bone fractures have become wide-spread major public health issues given the rapidly aging global population. Osteoporotic fractures are extremely harmful and are one of the primary causes of disability and death in elderly patients^2^. Within 1 year of experiencing a hip fracture, ~36% of patients may succumb to various complications, while about 50% may become disabled, leading to a significant decline in their quality of life^3^, and thus the disease constitutes a great international medical and economic burden^4^. In the US, in 2018, osteoporosis and low bone mass combined affected ~55.7% adults aged 50 and over^5^. In China, the estimated prevalence of osteoporosis is 20.73% among middle-aged and elderly men and 38.05% among middle-aged and elderly women^6^. And research has shown that if osteoporosis could be avoided then 1,364,717 of 2.7 million hip fractures that occurred in 2010 worldwide were potentially preventable^7^.

Although calculating the bone mineral density (BMD)-based T-score is the standard measure for the diagnosis of osteoporosis^8^, there is still a large degree of heterogeneity among cases of osteoporosis in evaluating its risk of fracture. This is because T-score values estimated by BMD from dual-energy X-ray absorptiometry (DXA) vary across DXA manufacturers^9^ and reference values^10^. Moreover, people diagnosed with osteoporosis have varying degrees of fracture risk^11^, suggesting that the diagnosis of osteoporosis needs to be more precise for each specific population. Several molecular markers, like bone turnover markers (BTMs), have been used recently to reflect the bone remodeling process and to measure the bone turnover rate of patients with osteoporosis. BTMs can also be used to monitor response to bone loss therapy, but they are not a satisfactory diagnostic index for identifying cases of osteoporosis^12^. Thus, there is an urgent unmet clinical need for new molecular candidate markers to more accurately identify patients at osteoporosis progression.

In the management of osteoporosis, calcium supplementation is widely recommended for the prevention of osteoporosis and fractures^11^. These recommendations are based on evidence from randomized controlled trials (RCTs) with BMD as the outcome, however, large meta-analysis and systematic review showed inconsistent treatment effects^13^. In a pooled study (7 prospective cohorts of 170,991 women, 5 prospective cohorts of 68,606 men, and 5 clinical trials of 6740 participants) suggested that calcium intake is not significantly associated with hip fracture risk^14^, yet other pooled studies had opposite results, considering calcium supplementation alone had a positive effect on bone density and a significant reduction in fractures^15^. Such studies indicate the role of calcium supplementation in the prevention of osteoporosis and fracture risk has not been well established, especially implicate inaccurate or imprecise diagnoses of osteoporosis and fractures could be an important reason for differences in drug efficacy.

The identification of osteoporosis and its subtypes with their corresponding molecular determinants is key for a personalized diagnosis and more effective treatment strategy. Along with the development of various high-throughput technologies, many kinds of omics data^16,17^ and integration with phenomics data^18,19^ provide the opportunity for osteoporosis stratification in a multi-omics manner. On the basis of epigenomics^20^, metabolomics and metagenomics^21^, some variants between normal bone and osteoporosis have been measured. However, large-scale studies connecting methylation, metabolism and microbiota to osteoporosis genomics are still lacking.

Here, utilizing 366 samples from the China Community-based Cohort of Osteoporosis (CCCO)^22^, we constructed the first prospective multi-omics atlas of the largest osteoporosis cohort to date. Such big biological data include genomics, epigenomics and metagenomics data for comprehensively characterizing the osteoporosis risk based on genetic and environmental factors, as well as metabolomics data for indicating the metabolic bone disorder and additional phenomics data for characterizing the observable covariates with osteoporosis risk. Next, we implemented an explainable data-intensive analysis framework for an omnigenic model^23,24^ based on a multi-modal approach, referred to as a deep latent space fusion (DLSF)^16,25,26^, which can help detect subtypes of osteoporosis with clinical significance and explain biological significance underlying osteoporosis and its subtypes with multi-modal molecular signatures (M3S). In addition, 2-year and 4-year follow-up outcomes were also collected to emphasize the clinical utility of the established osteoporosis and subtypes, which were also validated in another independent (Jinshan) cohort of osteoporosis with hundreds of samples.

**Fig. 1:**
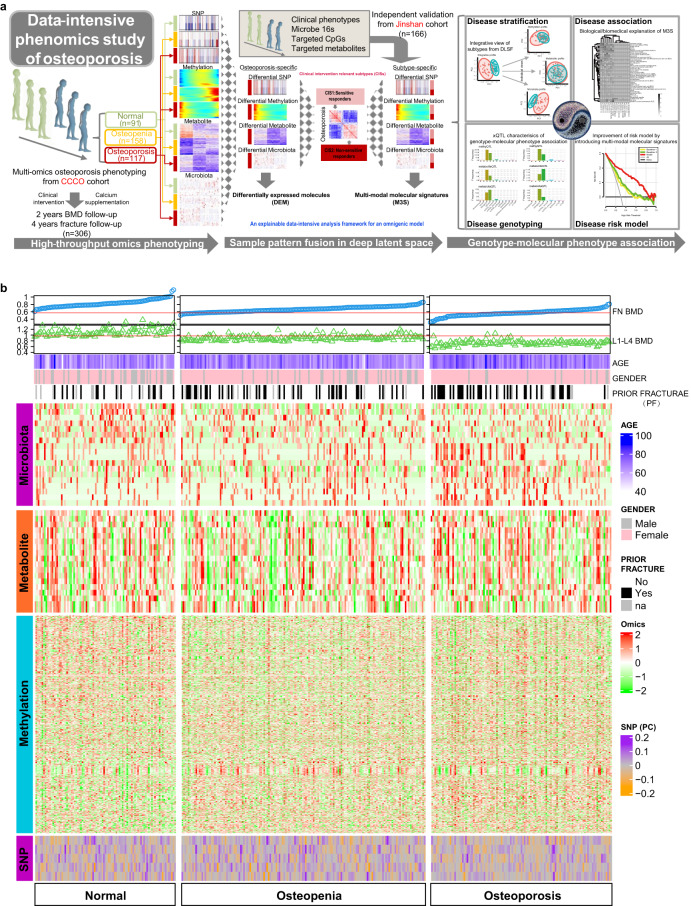
Design and workflow for studying osteoporosis and its subtypes on the basis of a multi-omics atlas generated by data-intensive analysis. **a** The study design and workflow in the principle of data-intensive analysis. **b** The global view of the molecular landscape of osteoporosis. The landscape consists of different data domains. (1) The BMD related indices such as FN BMD and TH BMD. (2) The clinical indicators such as age, gender and prior fracture. (3) The normalized expression of multi-omics including microbiota, metabolite and methylation CpG. (4) The global characteristics of SNPs as the top five principal components.

Through such a data-intensive study (Fig. 1a), we have firstly identified clinical intervention relevant subtypes (CISs) in a Chinese population based on the multi-omics landscape and corresponding M3S by efficient DLSF analysis, and CISs displayed different sensitivities during clinical intervention or prognosis (e.g., calcium supplementation). We then recognized many snpGenes associated with these molecular phenotypes by diverse candidate biological mechanisms underlying osteoporosis, and xQTL preferences indicate an omnigenic effect on different biological domains for explaining osteoporosis and CISs. Finally, the M3S as explicit functional representations of hidden genotypes can help develop improved osteoporosis risk models and identify new composite index for fracture prediction in clinical applications. Our integrative data resources and data-intensive analysis framework should greatly help future pre-disease and disease-onset studies in osteoporosis and relevant complex chronic non-communicable diseases, providing a road map to the development of precise early diagnostic standards, while accelerating discovery of novel drug targets and new prevention strategies.

## Results

### Multi-omics molecular landscape of osteoporosis

**Fig. 2:**
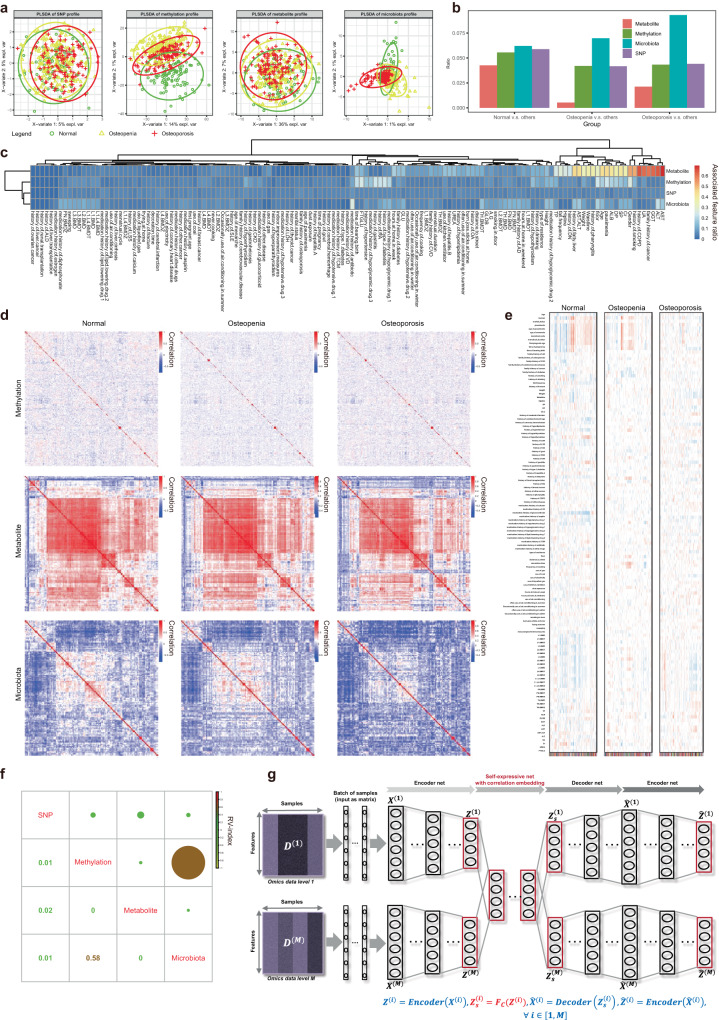
Characterization of multi-omics molecular landscape for osteoporosis. **a** Sample group distribution in the PLSDA space. **b** Percentages of discriminative features at different omics data levels. **c** Percentages of phenotype-associated features at different omics data levels. **d** Co-expression network of features for different sample groups at corresponding omics data level. **e** Co-expression network modules of features and their phenotype associations for different sample groups at particular omics data level. **f** Shared information between different omics datasets estimated by RV index. **g** The multi-omics unsupervised learning for osteoporosis multi-omics atlas by our deep latent space fusion model based on joint auto-encoder and self-expression technologies.

Based on biological high-throughput technologies, we have constructed the first prospective multi-omics molecular landscape of osteoporosis, by using genomics, epi-genomics, metabolomics and metagenomics data from matched samples within 117 osteoporosis individuals compared to 91 normal and 158 osteopenic individuals participated in the CCCO cohort (Fig. 1b). The demographic and clinical characteristics of the cohort participants were summarized in Supplementary Table S1. In each omics data modal, we observed different degrees of group discriminations: the methylation and microbiota modals showed more obvious separation between normal and disease groups than those of other modals (Fig. 2a), suggesting the necessity and importance of introducing molecular (microscopic) phenotypes (e.g., methylation, metabolites and microbiota) in addition to conventional genotypes (e.g., SNPs) to associate with physiological or pathological clinical (macroscopic) phenotypes. By differential expression analysis of each modal, microbiota as external factors had the highest percentage of relevant features detectable to distinguish osteoporosis from other groups, while SNPs and methylation as internal genetic factors showed a similar percentage of candidate discriminative features (Fig. 2b and Supplementary Tables S2–S5). Although metabolites tended to indicate the normal group (Fig. 2b), this modal was most efficient in detecting molecular associations with clinical phenotypes (Fig. 2c and Supplementary Table S6). For example, albumin, aspartate transaminase and alanine transaminase are associated with the crosstalk between osteoporosis and liver disease, while a history of smoking is known to be a risk factor for osteoporosis^27^, and uric acid has shown some protective effects on bone metabolism in Chinese postmenopausal females independent of body composition^28^. Thus, our multi-omics molecular landscape should help reveal the global and complete characteristics of osteoporosis.

In addition to these individual molecular features, the co-expression molecular networks and modules showed systematic and functional characteristics of osteoporosis and other osteopenia and normal groups. In general, the correlations among different molecular features were reduced from normal to osteopenia, and to osteoporosis (Fig. 2d), indicating the co-expression network coupling-separation underlying disease occurrence and progression. In particular, the co-expression modules identified by the typical WGCNA method^29^ displayed global trait association patterns for osteoporosis different to those for other groups (Fig. 2e and Supplementary Tables S7–S9).

The disease heterogeneity of osteoporosis has been widely observed with regard to the above molecular phenotypes. But in clinical practice, the diagnosis of osteoporosis is mainly based on BMD measurements via DXA measurements. This “one size fits all” approach is poorly equipped to explain the complex disease status and treatment response of osteoporosis observed in the clinic. Thus, greatly inspired by the molecular clues above, we sought to apply our multi-modal approach to identify potential disease stratifications in an effort to better understand the disease heterogeneity of osteoporosis and guide clinical determinations that may lead to a higher quality of care. To this aim, we sought to identify subtypes of osteoporosis in an integrative manner, while providing multi-modal molecular signatures (M3S) for osteoporosis diagnosis and fracture risk evaluation. By a theoretical estimation, the information fusion or sharing among different omics data has to be measured with an RV index^30^. The quantitative values remarkably supported the notion that the multi-omics molecular landscape supplied complementary information in a combinatorial manner (Fig. 2f). For example, the methylation and microbiota datasets tended to have greater overlap or explained information, whereas any other two datasets had different or independent information.

Supporting these requirements and motivation, our previously developed deep learning model, Deep Latent Space Fusion (DLSF), has shown an efficient analysis capability in multi-omics data integration for cancer subtypes^16^. Thus, DLSF, as a key component of our data-intensive analysis pipeline (Fig. 1a), was used to identify the molecular stratification of osteoporosis individuals and corresponding subtyping signatures undetectable in typical DXA analysis, based on their multi-omics molecular landscape (Fig. 2g), and whose sample network in latent data space rather than in observation data space allowed us to identify sample clusters (i.e., osteoporosis subtypes) shared in the multi-modal information.

### The identification of osteoporosis subtypes via the multi-omics molecular landscape by DLSF

**Fig. 3:**
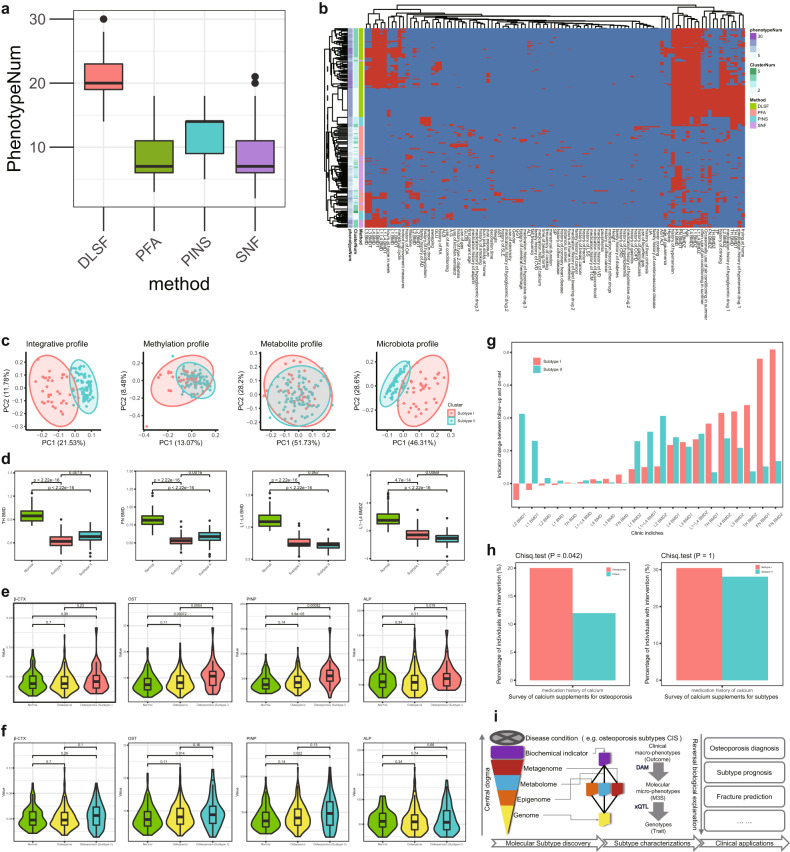
Multi-modal subtypes of osteoporosis identified by deep latent space fusion model. **a** Efficiency evaluation of DLSF and other models based on the number of phenotype associations. **b** Robustness evaluation of DLSF and other models. **c** Integrative osteoporosis subtypes and their discriminative spaces shown in different omics data level. **d** Several phenotypes relevant to osteoporosis and two identified subtypes (Subtype I and Subtype II), including several BMD related indices such as TH BMD, FN BMD, L1–L4 BMD, and L1–L4 BMDZ. Subtype I tends to have smaller TH BMD and FN BMD and larger L1–L4 BMD and L1–L4 BMDZ compared to Subtype II. **e**, **f** BTMs difference between normal, osteopenia and osteoporosis Subtype I and Subtype II respectively. **g** Two-year follow-up evaluation of Subtype I and Subtype II. **h** Survey of medical history for osteoporosis, indicating the percentage of individuals receiving particular medical intervention (e.g., calcium supplements), and their distribution differences between osteoporosis and others, or between two osteoporosis subtypes. **i** The analysis workflow for osteoporosis and its subtypes. After subtype discovery through efficient DLSF, DAM and xQTL can provide subtype characterizations in the manner of reversal biological explanations, based on which osteoporosis risk models and fracture risk assessment can be further established for clinical applications.

DLSF allowed us to identify sample clustering associated with more clinical phenotypes than those by other conventional multi-omics analysis methods, including Similarity Network Fusion (SNF)^31^ and Perturbation clustering for data INtegration and disease Subtyping (PINS)^32^ (Fig. 3a). It also detected more balanced clusters (e.g., different clusters with similar number of samples) than those by PINS (Supplementary Fig. S1). DLSF is an efficient approach to analyze our multi-omics molecular landscape of osteoporosis. The sample clustering is robust for DLSF, and we were able to determine two clusters using this approach, as we considered the cluster balance and association to be relevant to clinical phenotypes (Fig. 3b and Supplementary Table S10).

According to such subtype determined by molecular phenotypes, osteoporosis individuals could be clearly divided into two groups in the unified latent data space (Fig. 3c and Supplementary Tables S11 and S12), and the same samples showed similar groups in different individual observation data spaces, especially in microbiota and methylation modals. The two sample clusters/subtypes had strong associations with many clinical phenotypes (Fig. 3d and Supplementary Figs. S2 and S3). For example, they displayed many phenotype distribution differences between two subtypes. Notably, individuals within Subtype I tended to have lower total hip bone mineral density (TH BMD) and other related indices like lower femoral neck bone mineral density (FN BMD) compared to Subtype II. Meanwhile, individuals within Subtype II had lower lumbar spine bone mineral density (L1–L4) (L1–L4 BMD) and other indices like a lower L1–L4 BMD *Z*-score.

The two subtypes also showed differential changes in BTMs (Fig. 3e, f). Subtype I had significant changes in N-terminal propeptide of type I procollagen (PlNP) and osteocalcin (OST) compared to the normal and osteopenic groups; meanwhile, Subtype II had significant changes on PlNP and OST compared to the normal group. Of note, Subtype I had certain differences in alkaline phosphatase (ALP), and the two subtypes had a changed trend and varied distribution of β-CrossLaps of type I collagen containing crosslinked C-telopeptide (β-CTX). These results showed that Subtype I and II both display dysfunction of BTMs. Especially, these osteoporosis subtypes were evaluated for their responses to therapy by follow-up BMD outcomes of the same individuals from the CCCO cohort. Actually, many clinical indices of BMD were improved for Subtype I compared to those of Subtype II (Fig. 3g). This strongly supports the notion that such new osteoporosis subtypes have a remarkable clinical relevance and that the Subtype I has a strong disposition for BMD improvement against osteoporosis, which would be the sensitive subgroup for clinical intervention of the disease. Thus, these subtypes have remarkable clinical intervention relevance, and could be annotated as clinical intervention relevant subtypes (CISs) instead of original terms of Subtype I/II. In addition, according to our survey of medical history, we observed that the percentages of individuals receiving calcium supplements are significantly different between osteoporosis and others, rather than between osteoporosis subtypes (Fig. 3h and Supplementary Fig. S4). This fact indicated that osteoporosis individuals can be improved by taking necessary calcium supplements, and different osteoporosis subtypes would have different sensitivities during such improvement process, e.g., CIS1 (Subtype I) is a sensitive group while CIS2 (Subtype II) tends to be a less-sensitive or non-sensitive group.

These osteoporosis subtypes were efficiently identified by DLSF, an upstream method for discovering molecular subtypes consistent with biological central dogma (Fig. 3i). Therefore, the next downstream procedure is to characterize the subtypes (i.e., CISs) through a reverse biological explanation (Fig. 3i), which mainly includes the Differential Association Matrix Analysis (DAM) to characterize the phenotype-relevant molecular signatures and the Multi-Omics Quantitative Trait Locus (xQTL) to characterize the phenotype-relevant genotypes, and to implement clinical applications in osteoporosis diagnosis, subtype prognosis, fracture prediction and so on (Fig. 3i).

### Molecular signatures of the osteoporosis subtypes among different modals

**Fig. 4:**
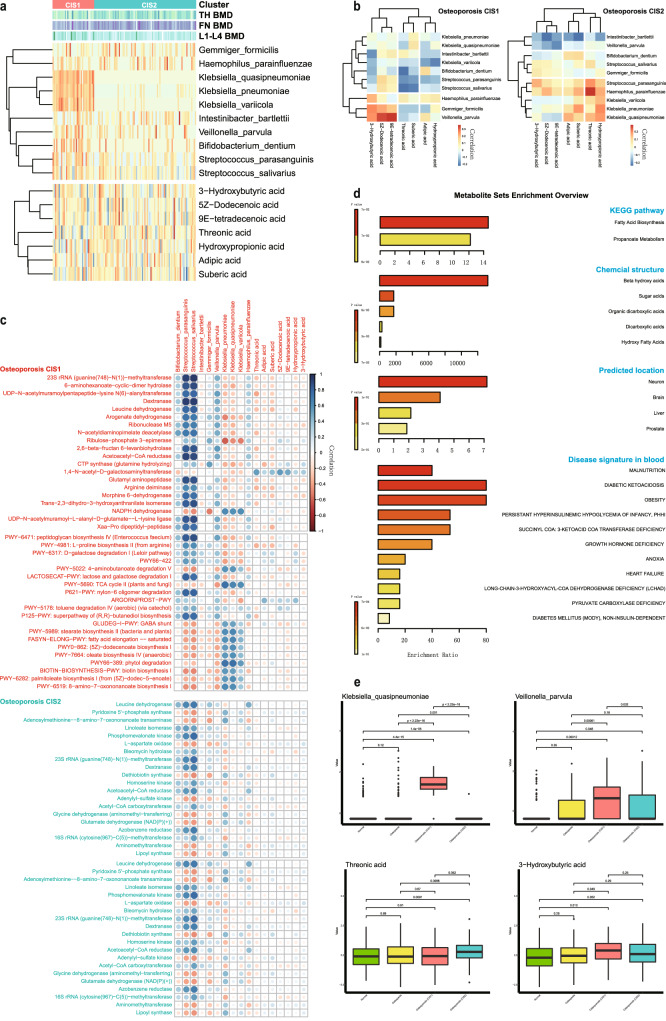
Microbiota and metabolite signatures associated with osteoporosis and its subtypes. **a** Expression profiles of microbiota and metabolite signatures during two osteoporosis subtypes. **b** Association matrix among microbiota and metabolite signatures during two osteoporosis subtypes. **c** Subtype-specific association matrix among microbiota/metabolite signatures and annotated functions and pathways. **d** Functional enrichments of metabolite signatures. **e** Several representative microbiota and metabolite signatures, indicating osteoporosis and its subtypes.

To understand the molecular characteristics underlying the identified osteoporosis subtypes, the molecular signatures of M3S for each modal were investigated. Firstly, the microbiota and metabolite signatures of M3S were extracted as microbiomes are thought to crosstalk and have functional impacts on host metabolism^33^. Microbiota showed greater discrimination among subtypes than metabolite (Figs. 4a and 2a), suggesting its dominant efficiency in our analyses. The microbiota signatures have rewiring associations with many metabolite signatures for different subtypes (Fig. 4b). For example, the correlation between *Veillonella parvula* and 3-hydroxybutyric acid is positive in CIS1 while negative in CIS2 (Fig. 4b). There are many case reports regarding the contribution of *Veillonella parvula* to various bone diseases, including spondylodiscitis, osteomyelitis and primary sclerosing cholangitis^34–36^. Recent studies have further suggested an association between osteoporosis and these bone dysfunctions. For example, pyogenic spondylodiscitis is able to cause severe osteolytic and destructive lesions^37^, while osteomyelitis and osteoporosis can both lead to loss of bone mass, where infections can alter RANKL-RANK-OPG signaling that is involved in the regulation of osteoblast and osteoclast behavior^38^. Furthermore, osteoporosis is an oft-occurring bone disease in cholestatic liver diseases, like primary biliary cholangitis and primary sclerosing cholangitis^39^.

Regarding the microbiota signature, we also found the association between *Klebsiella quasipneumoniae* and threonic acid is negative in CIS1 but positive in CIS2 by DAM (Fig. 4b). *Klebsiella pneumoniae* has a similar metabolism association to *Klebsiella quasipneumoniae*, and it has been shown to induce the generation of inflammatory immune cells that can migrate to distant inflammatory tissues in the context of a genetically susceptible host where it takes part in the onset and progression of non-intestinal inflammatory diseases, such as arthritis^40^. In addition, the concentration of 3-hydroxybutyric acid is elevated in individuals with type I diabetes and diabetic coma^41^, and threonic acid is involved in calcium absorption^42^, indicating their potential discriminative roles and functional relevance in osteoporosis and subtypes.

The common and specific roles of these signatures in osteoporosis and its subtypes were also supported by their associations with osteoporosis-related functions (Fig. 4c). In general, microbiota signatures showed more functional associations than metabolite signatures, indicating the common and specific metabolic dysfunctions caused by microbes and host. We also found that these signatures had more functional associations in CIS1 than in CIS2, suggesting again the relative functional rewiring that occurs between these two subtypes of osteoporosis. Meanwhile, in contrast to *Klebsiella quasipneumoniae*, *Streptococcus salivarius*, *Streptococcus parasanguinis* and *Bifidobacterium dentium* displayed certain consistent functional associations in the two subtypes, which have been reported to be involved in schizophrenia^43^, and it should be noted that schizophrenia is thought to be correlated to bone fragility^44^. On the other hand, metabolite signatures revealed additional pathogen information (Fig. 4d). By enrichment analysis, these metabolites mainly belong to beta hydroxy acids, which act in fatty acid biosynthesis and propanoate metabolism. There are predicted functional locations, including in neurons, the brain and the liver. These metabolites are also involved in many diseases, including obesity, diabetes, growth hormone deficiency and pyruvate carboxylase deficiency. Similar to osteoporosis, type 2 diabetes is also affected by aging and the two diseases often coexist. Thus, fracture risk is actually increased in patients with diabetes^45^. In addition, untreated growth hormone deficiency may cause a higher risk of vertebral fractures in adult patients^46^.

Supported by the above diverse functional evidence, the microbiota and metabolite signatures are explainable and efficient biomarker candidates for indicating osteoporosis and its subtypes (Supplementary Fig. S5). For example, compared to the normal and osteopenia groups, *Klebsiella quasipneumoniae* and *Veillonella parvula* had specific expression in osteoporosis CIS1, and 3-hydroxybutyric acid tended to be upregulated in osteoporosis CIS1, while, in contrast, threonic acid was upregulated in osteoporosis CIS2 (Fig. 4e). In addition, the similar microbiota signatures on the genus level could also be screened, which can be efficiently and alternatively applied when the 16S data are available. Indeed, we found them to be consistent to the above findings and included such genuses as *Klebsiella*, *Veillonella*, *Bacteroides* and *Streptococcus* (Supplementary Fig. S6).

Although osteoporosis is known as a metabolic disease, recent studies have shown that abnormal epigenetic regulation (e.g., aberrant DNA methylation) can also cause bone metabolism-related dysfunction on osteogenic differentiation, osteogenesis and bone remodeling, which should provide a new understanding on the pathogenesis of osteoporosis^47^ and potential treatment targets of the disease^48^.

**Fig. 5:**
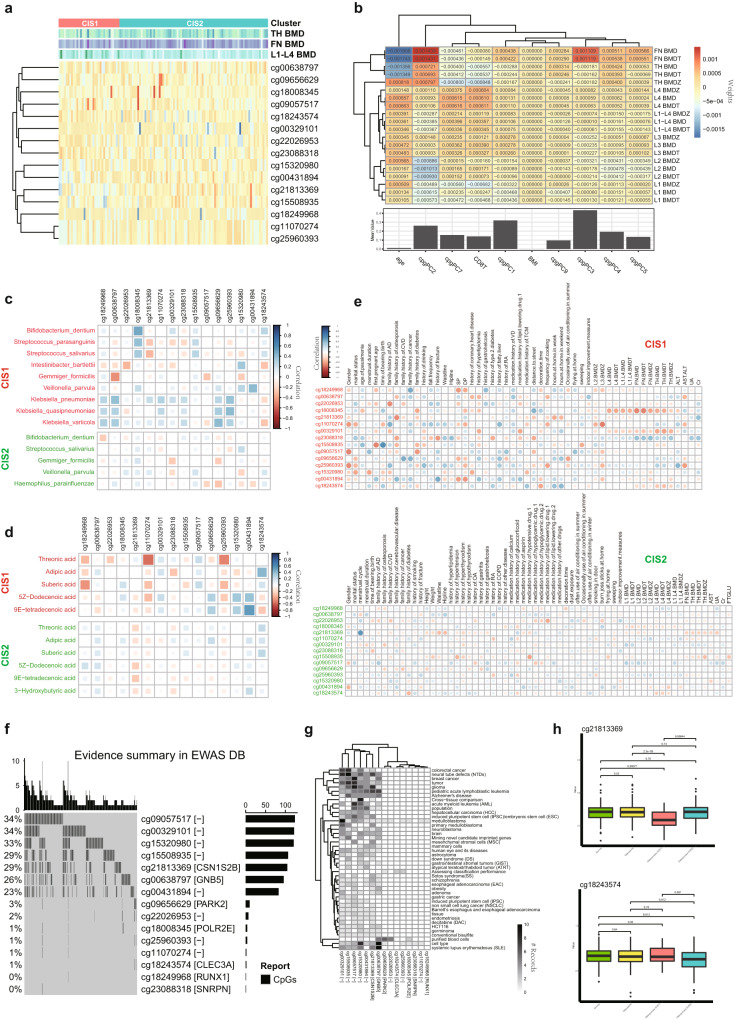
Methylation signatures associated with osteoporosis and its subtypes. **a** CpGs of methylation signatures. **b** Summary of EWAS results. **c** Subtype-specific association matrix among methylation and microbiota signatures. **d** Subtype-specific association matrix among methylation and metabolite signatures. **e** Subtype-specific association matrix among methylation signatures and clinical phenotypes. **f** Public reports about methylation signatures in EWAS DB. **g** Public reported phenotypes associated with methylation signatures. **h** Several representative methylation signatures, indicating osteoporosis and its subtypes.

In the methylation modal of our multi-omics atlas, a general epigenome-wide association study (EWAS) analysis confirmed the contribution of epigenetic information to different clinical phenotypes (Fig. 5a). Compared to age or body mass index (BMI), different principal components (PCs) of the methylation profile tended to have many effects on the genotype-phenotype associations, and different PCs showed dissimilar preferences. For example, cpgPC2 and cpgPC3 had a greater association with FN BMD while cpgPC7 had a greater association with L1–L4 BMD, compared to the average of all phenotypes (Fig. 5b). These findings suggest that the methylation profile is capable of providing common and specific information and features to define osteoporosis and its subtypes.

Our analysis had screened out a set of CpG sites related to osteoporosis and its subtypes (Fig. 5a). These methylation signatures have corresponding associations with the above metabolite and microbiota signatures (Fig. 5c, d), indicating their potential relevance to metabolic alterations during osteoporosis. For instances, cg18008345 showed a positive correlation with *Streptococcus salivarius* and *Streptococcus parasanguinis* in CIS1 but a reduced correlation in CIS2, while cg09057517 had a negative correlation with *Gemmiger formicilis* in CIS1 and with *Haemophilus parainfluenzae* in CIS2 (Fig. 5c). Furthermore, cg18243574 displayed many associations with microbiota signatures, including *Klebsiella pneumoniae*, in CIS1, while cg18249968 had a remarkable negative correlation with *Bifidobacterium dentium* in CIS2 (Fig. 5c). These results reveal that host epigenetic information can explain many common and specific features observed in external factors, like the microbiota. Similarly, these methylation signatures showed subtype-specific correlations with metabolite signatures, including adipic acid, 5Z-dodecenoic acid and 9E-tetradecenoic acid, indicating the complementary information and representation on epigenetic and metabolomic levels (Fig. 5d). Of note, cg18249968 was negative with most metabolite signatures in CIS1, but positive with them in CIS2.

These cross-modal associations by DAM suggest that methylation signatures must also have common and specific associations with clinical phenotypes (Fig. 5e). Our analysis found a few shared associations, including that cg18008345 was remarkably negatively associated with L1–L4 BMD indices in different subtypes, and that cg23088318 was positively associated with them, while cg21813369 illustrated negative correlations with many indices in two subtypes (Fig. 5e). Meanwhile, we also observed subtype-specific associations, including that cg09057517 was positively associated with a history of smoking in CIS2, that cg18243574 in CIS1 had a negative correlation with a family history of diabetes and a positive correlation with medication history of traditional Chinese medicine (TCM), and that cg18249968 and some other sites had negative correlations with systolic pressure (SP) and diastolic pressure (DP) in CIS1 rather than those in CIS2.

Notably, these methylation signatures were detectable in public EWAS studies, including EWASdb^49^, and many signatures locate with methylation-regulated genes on DNA sequences, which have functional relations with osteoporosis (Fig. 5f and Supplementary Table S13). For example, cg18008345 is a site within *POLR2E*. This gene is upregulated during osteoclastogenesis, and its inhibition decreases osteoclastogenesis while its overexpression shows completely opposite effects in vitro, whose gene product interacts with CREB1 to regulate osteoclastic bone resorption^50^. cg21813369 is located within the *CSN1S2B* locus, and in bones of type 2 diabetic rats, CSN1S2 protein has shown a protective effect against osteoporosis via bone morphometric protein signaling^51^. cg18243574 is a site within *CLEC3A*, and this gene is a member of the C-type lectin superfamily and a candidate oncogene in osteosarcoma, whose suppression might inhibit osteosarcoma cell proliferation and promote chemosensitivity^52^. cg18249968 is localized to *RUNX1*, which is highly expressed in osteoblasts and *RUNX1* maintains adult bone homeostasis from bone loss, which could be a new therapeutic target for osteoporosis^53^. cg09656629 is localized to *PARK2*, whose variations have been linked to rare, inherited forms of Parkinson’s disease^54^, and osteoporosis and Parkinson’s disease often co-occur, indicating a possible similar role for *PARK2* variations in osteoporosis^55^.

We also filtered out many osteoporosis-related phenotypes to link our targeted sites and genes (Fig. 5g and Supplementary Fig. S7). For instance, the CpG variants in the following genes have reports to many osteoporosis-related phenotypes: *CSN1S2B* and *GNB5* are associated with obesity, schizophrenia and osteosarcoma, *PARK2* is linked to Crohn’s disease with known influencing factors of osteoporosis, and *RUNX1* is associated with medulloblastoma, whose most common sites of extraneural metastases include skeleton and bone marrow^56^.

Given these functional relevance and evidence, we confirmed the biological and biomedical significance of the methylation signatures of M3S, whose expressions demonstrated significant down-regulated changes in identified osteoporosis subtypes (Fig. 5h and Supplementary Fig. S5). cg21813369 (CSN1S2B) had lower expression in osteoporosis CIS1 compared to the normal and osteopenia groups. In contrast, cg18243574 (CLEC3A) had lower expression in osteoporosis CIS2. These signatures represent molecular characteristics of new osteoporosis subtypes at the epigenetic level.

### Genotype associations underlying multi-modal molecular signatures of osteoporosis subtypes

The above osteoporosis subtypes were defined and characterized by integrative molecular phenotypes, e.g., represented by M3S. The genotypes associated with these signatures of molecular phenotypes are expected to further reveal potential common and specific genetic determinants relevant to osteoporosis and its subtypes.

**Fig. 6:**
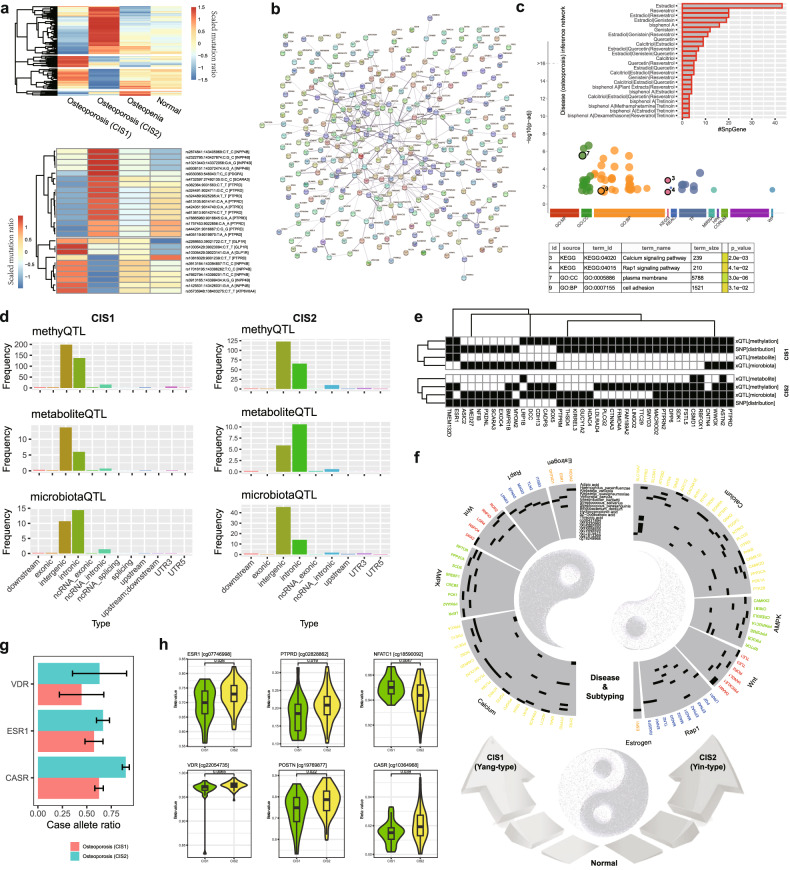
Genome characteristics underlying multi-modal molecular signatures M3S of osteoporosis subtypes in a Yin-Yang scheme. **a** Global SNV patterns of osteoporosis subtypes. **b** Protein association network among global snpGenes. **c** Functional enrichment of global snpGenes. **d** Preferences of SNV types of xQTL SNPs corresponding to different osteoporosis subtypes. **e** Shared and specific local snpGenes among different QTLs from xQTL model. **f** Yin-Yang osteoporosis subtypes characterized by the subtype-specific genotype-molecular phenotype associations. **g** Allele frequency of representative Calcium related genes. **h** Methylation differences between CISs, with the cases of a few Calcium and osteoporosis-related genes.

On the one hand, the differences in SNP/SNV distribution in the normal, osteopenia and osteoporosis groups were summarized (Fig. 6a; Supplementary Fig. S8 and Table S14), and we found two subtypes of osteoporosis that had remarkable changes in global SNP patterns compared to the other groups. These findings suggest the genotype changes that exist in osteoporosis and its subtypes. Multiple SNPs located at a global snpGene *INPP4B* tended to have consistent changes in CIS1, which are similar for *ATP6V0A4* and *GLP1R*. Several SNPs located at another global snpGene *PTPRD* had consistent changes in CIS2, where *PDGFA* and *SCARA3* had similar changes. *INPP4B*^57^ and *ATP6V0A4*^58^ are known modulators of osteoclast differentiation and have a prognostic locus for human osteoporosis, while *SCARA3* is known to regulate the switch between adipocyte and osteoblast differentiation and thus represents a potential therapeutic target for bone loss and osteoporosis^59^. These results indicate diverse causality of SNPs on molecular phenotypes. Most global snpGenes showed dense functional protein interactions or associations (Fig. 6b) and were enriched in the Calcium signaling pathway and Rap1 signaling pathway (Fig. 6c). Calcium signaling participates in the differentiation and function of osteoclasts, whose dysfunction can cause the abnormalities in osteoclastic bone resorption involved in osteoporosis^60^. Rap1 signaling targets are known to participate with epigenetic factors in the modulation of osteogenesis by coordinating osteoblast/osteoclast differentiation^61^. And Rap1 is known to play critical roles in the resorptive function of osteoclasts, and its selective inhibition in mature osteoclasts retards pathological bone loss^62^. Notably, many global snpGenes were reported in The Comparative Toxicogenomics Database (or as CTD genes)^63^, and they have pathogenic roles in many osteoporosis-relevant inference networks, including Estradiol and Resveratrol (Fig. 6c and Supplementary Fig. S9). These findings dovetail with previous findings that older men with total estradiol deficiency are osteoporotic and those with osteoporosis are more likely to be total estradiol deficient^64^.

On the other hand, the xQTL models^65^ corresponding to two osteoporosis subtypes were investigated to infer the quantitative association between genotype and molecular phenotypes (e.g., M3S). From the distribution of QTL SNP variant types, we indeed observed again the common and specific molecular characteristics for the two subtypes (Fig. 6d). For the genotype features underlying M3S with quantitative associations, the dominate distributions of variants are intergenic and intronic, which were consistent for different types of QTLs, including methyQTL, metaboliteQTL and microbiotaQTL. In contrast, the specific characteristics of SNP variant types were also observed and compared to CIS2, CIS1 had greater intronic variants in microbiotaQTL and less intronic variants in metaboliteQTL. Indeed, such local snpGenes involved in xQTL had many overlaps with those in the above global snpGenes (Fig. 6b). By comparison, CIS1 had the most similar snpGenes between methyQTL and global ones, while CIS2 had the most similar snpGenes between microbiotaQTL and global ones (Fig. 6e). These factors supported again the necessity and importance of multi-omics analysis and multi-modal model for osteoporosis.

Subtypes of osteoporosis could be greatly reflected by different molecular phenotypes. Of note, snpGenes *PLCG2*, *FRMD4A* and *MED27* were consistently found in most kinds of genotype-phenotype associations in CIS1 and belonged to CIS1-specific global snpGenes. Meanwhile, *RBFOX1* and *ASTN2* were consistently found in all kinds of genotype-phenotype associations in CIS2 and were CIS2-specific global snpGenes. Furthermore, SNPs in *ESR1* and *CSMD1* had genotype-phenotype associations with the two subtypes and showed consistent variant distributions corresponding to subtype-specific global snpGenes. There are many reports regarding the roles of *ESR1* and *ESR2* polymorphisms in osteoporosis, which have demonstrated potential heterogeneity associated with osteoporosis risk^66^. *CSMD1* is a schizophrenia-associated gene and there is a potential risk of osteoporosis in schizophrenia^67^. By enrichment analysis, several key functions related to osteoporosis subtypes were further confirmed (Supplementary Fig. S10). The Calcium signaling pathway was enriched in CIS2-related xQTL snpGenes, while nitric oxide stimulates guanylate cyclase was enriched in CIS1-related xQTL snpGenes. As previously reported, vasculature-produced nitric oxide is essential for the mobilization of stem and progenitor cells, which can arise from the bone marrow stem cell niche^68^, and nitric oxide can cooperate with AMPK in the regulation of skeletal muscle cells^69^.

Notably, the analysis of typical osteoporosis-associated signaling pathways inspired our findings to link genotype and phenotype (Fig. 6f and Supplementary Tables S15 and S16). CIS1 had relatively more xQTL associations with genes that participate in estrogen and AMPK signaling pathways, which was a group of sensitive responders in osteoporosis individuals, and annotated as Yang-type representing an expectation of great improvement in BMD during clinical intervention. In contrast, CIS2 had genotype-molecular phenotype associations with a great number of genes involved in the calcium signaling pathway, which was a group of non-sensitive responders in osteoporosis individuals, and annotated as Yin-type indicating a low expectation of BMD improvement.

The above clinical and molecular evidence of osteoporosis subtypes indicated the strong contribution of calcium relevant functions and potential subtype-specific responses. As reported, the “non-responders” to calcium and vitamin D supplementation have higher frequency of polymorphisms in the ESR1 and VDR^70^, compared to “responders”. Indeed, we also consistently found that CIS2 tend to have higher allele frequency of SNVs on these genes, and some new relevant genes like calcium-sensing receptor (CASR), compared to CIS1 (Fig. 6g and Supplementary Table S17), suggesting the candidate genetic determinants of sensitivity difference on calcium supplementation between CIS2 and CIS1. In addition, we also found many candidate epigenetic determinants of such sensitivity difference, and many CpGs of Calcium relevant genes displayed hyper-methylation in CIS2 (Fig. 6h and Supplementary Fig. S11). For example, ESR1, VDR, CASR and the osteoblasts related gene BMPR1B would have hyper-methylations in CIS2 compared to in CIS1. By contrast, the osteoclast related genes NFATC1 and HDAC4^71,72^ might be hyper-methylation in CIS1 (Fig. 6h and Supplementary Fig. S12). This certainly explains the sensitivity of CIS1 individuals receiving calcium supplementation, and the insensitivity of CIS2 compared to CIS1 due to inhibition of calcium signaling pathway by epigenetic factors.

### Improved discrimination of osteoporosis and its subtypes by introducing M3S into the risk model

**Fig. 7:**
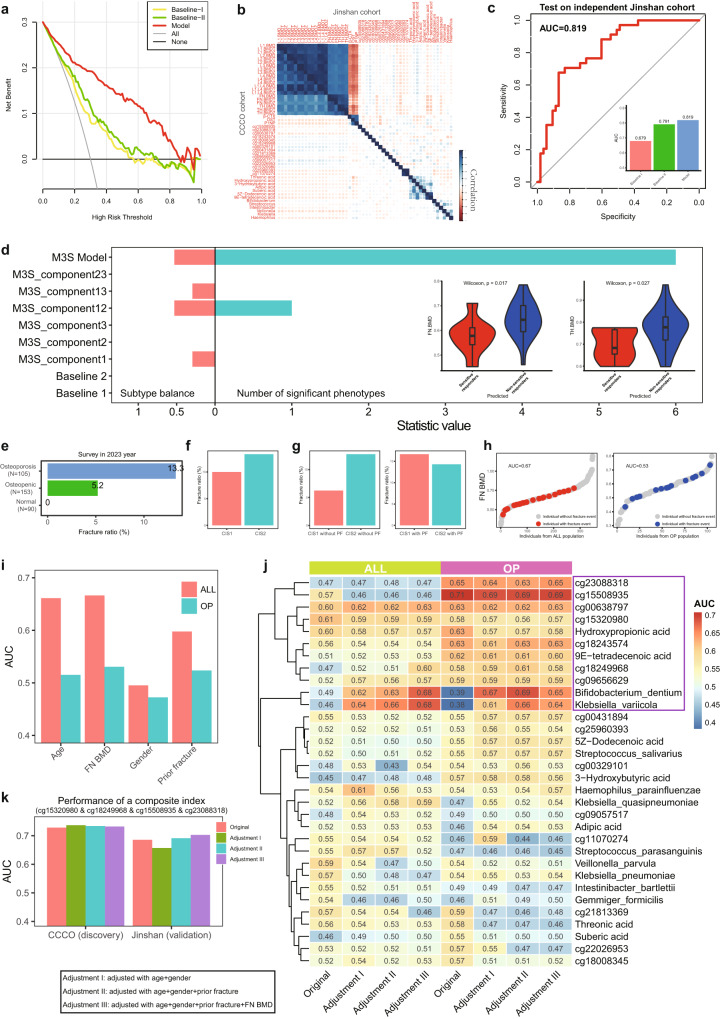
Clinical application of M3S including risk models for osteoporosis and fracture risk assessment. **a** Decision curve of different risk models (osteoporosis vs others), Baseline I: conventional risk factors; Baseline II: Baseline I + BTMs; Model: Baseline II + our multi-modal molecular signatures (M3S). **b** Independent evaluation of phenotype associations between the CCCO and Jinshan cohorts. **c** Independent performance evaluation of risk model (osteoporosis vs others) based on M3S, compared to two baseline methods. **d** Independent performance evaluation of risk model (Yang vs Yin subtypes) based on M3S, compared to other component models and baseline methods. **e** Statistic of fracture outcome of the CCCO cohort at 4-year of follow-up. **f** The fracture rate of different osteoporosis subtypes. **g** The fracture rate of different osteoporosis subtypes adjusted by prior fracture (PF). **h** Cases of fracture risk assessment by ranking measured with AUC, e.g., the AUC of FN BMD was evaluated in the ALL population (including normal, osteopenic and osteoporosis individuals) and OP population (including individuals with osteoporosis only) respectively. **i** The AUC of traditional clinical indices of fracture risk. **j** The original and adjusted AUC of each M3S feature in the ALL and OP populations respectively. **k** The evaluation of a composite index for fracture risk assessment according to original and adjusted AUC for the discovery cohort (CCCO) and for the independent validation cohort (Jinshan) respectively.

There are many analyses demonstrating the biological foundation of M3S related to osteoporosis events and many clinical phenotypes, and thus they should improve the risk model based on BTMs with necessary covariates, like age and BMI^12^. Here, three kinds of logistic regression (LR) risk models were constructed on hundreds of normal and osteoporosis samples. The first is Baseline I, which is built on age, BMI and histories of drinking and smoking. The second is Baseline II, which is Baseline I + BTMs (i.e., β-CTX, OST and PINP). The third is our new Model, which is Baseline II + M3S. According to the decision curves of our analysis data, our new Model outperformed the other two baseline models in diagnosing osteoporosis (Fig. 7a), indicating the improved clinical discrimination introduced from M3S. Since the risk of osteoporosis may differ between women and men, it is worth noting that gender could also be included in the relevant risk models.

Next, we have validated M3S, BTMs and their associations in an independent (Jinshan) cohort with hundreds of normal and osteoporosis samples, where 87 samples have matched multi-omics data. We found that M3S indeed had consistent associations in the new cohort, especially for microbiota and metabolite modal (Fig. 7b and Supplementary Figs. S13–S15), which again suggested the molecular heterogeneity of osteoporosis. Thus, the risk model based on these two modal was re-trained again after correction of potential batch effects existing in omics data (Supplementary Figs. S16–S19), and it still achieved a higher performance than the two baseline models (Fig. 7c and Supplementary Figs. S20 and S21); notably, with an independent validation AUC of 0.819.

On the other hand, another M3S-based risk model for subtype evaluation can efficiently recognize potential subtype individuals in the independent Jinshan cohort (Fig. 7d). In contrast, the baseline models and M3S component models without methylation information tended to have prediction bias (e.g., assessed by balance index) and predicted all individuals to be the same subtype, which suggested the importance and necessity of multi-omics information and multi-modal modeling for detecting and characterizing osteoporosis subtypes. Similarly, there were many phenotype associations with the individual subtype predicted by the M3S model than those of other methods (e.g., assessed by number of associated phenotypes) (Fig. 7d and Supplementary Fig. S22), as especially the associations with FN BMD or TH BMD in the Jinshan cohort were consistent to those found in CCCO cohort (Fig. 3d).

If gender is included in the risk models above, we could actually achieve some improvements in prediction (Supplementary Fig. S23), which should be more valuable if the model is built on a larger cohort with a balanced number of male and female individuals.

### Risk of fragile fracture assessed by M3S for osteoporosis

BMD is thought to be associated with fracture outcome. The lower the BMD, the higher the risk of fracture^73,74^. When tracking fracture events of our cohorts collected in 2023, we found that the osteoporosis group actually had a higher ratio of fractures than normal groups (Fig. 7e), supporting the causal relationship between BMD and fracture. Meanwhile, CIS1 individuals have lower fracture ratio than CIS2 individuals across osteoporosis subtypes. It is clinically accepted that people who have fractures have a higher chance of breaking again. In fact, we observed that CIS1 individuals without PF (Prior Fracture) have the lowest fracture ratio compared to other conditions; in contrast, CIS2 individuals have a fracture ratio greater than 10% regardless of whether PF is taken into account or not (Fig. 7f, g and Supplementary Fig. S24a). Thus, regardless of whether the molecular mechanisms of CIS are calcium sensitive or not, the role of prior fracture as a risk factor cannot be overemphasized, which better illustrates the existence of different molecular mechanisms for CIS1 and CIS2.

It is possible to evaluate biomarkers for predicting the fracture risk by sorting the indicator values. Taking FN BMD as an example (Fig. 7h), the values of different individuals can be arranged in ascending order (e.g., gray dots) and the fracture event can be labeled (e.g., red dots). Apparently, in the statistics of the entire population (i.e., ALL group including normal, osteopenic and osteoporosis individuals), there is indeed a possibility that individuals with lower BMD will experience fracture events. In contrast, in the OP population (i.e., OP group comprising individuals with osteoporosis), the distribution of fracture events among BMD values is scattered, suggesting that risk assessment of fracture prediction in osteoporosis would be a new clinical prognostic problem where BMD would make a smaller contribution/association for the occurrence of fractures. Based on further quantitative assessment of predictive power using Area Under Curve (AUC), FN BMD actually had high predictive power in the ALL population (AUC = 0.67), while the AUC in the OP population was lower to 0.53 (Fig. 7h). Indeed, other previously reported clinical indicators^75^ associated with fractures also supported this conclusion (Fig. 7i).

Therefore, we used the same AUC measurement to assess the predictive power of fracture risk for each omics feature (Supplementary Fig. S24b), e.g., methylation site, metabolite and microbiota. On the one hand, ~2 K candidate indicators (AUC > 0.6) were found for both ALL and OP populations, with an intersection of about 50%, again confirming that osteoporosis has remarkably specific molecular factors for fracture risk. On the other hand, as previously reported, BMD is associated with fractures in ALL population, which may serve as covariates for the above-mentioned omics features. Thus, we adjusted the AUC score of each omics feature using three methods (Supplementary Fig. S24b): (1) age + gender; (2) age + gender + prior fracture; and age + gender + prior fracture + FN BMD. In fact, there were about 20% of omics features with a significant decrease in AUC after correction, but most omics features still had a high AUC of > 0.6. Accordingly, there was no significant association between BMD and fractures in OP population, which could be a confounding factor. Therefore, after adjustment, especially after BMD correction, we were able to detect many osteoporosis-specific fracture risk factors. These results again suggested that omics features may provide more osteoporosis-specific and BMD-independent fracture risk factors in contrast to traditional clinical indicators.

Especially, M3S can actually provide effective osteoporosis-specific biomarkers of fracture risk (Fig. 7j). For example, methylation sites such as cg23088318 (AUC > 0.65) and cg155008935 (AUC ~ 0.7) are osteoporosis-specific. Meanwhile, *Bifidobacterium Dentium* and *Klebsiella Variicola* would be potential biomarkers for both ALL and OP populations regardless of BMD. As is known, the combination of different biomarkers would achieve some predictive support. Given that the methylation sites in M3S showed remarkable predictive power for fracture risk, we screened the entire combinations of a small panel of CpGs from M3S (*N* < 5) and found some candidate composite indices, quantified by a simple sum of member CpGs’ abundances. For example, the combination of cg15320980 & cg18249968 & cg15508935 & cg23088318 could achieve an AUC of 0.73 in the discovery cohort (CCCO), and even a validation AUC of 0.7 in the independent Jinshan cohort (Fig. 7k).

## Discussions

Compared to individual-omics studies^76^, multi-omics analyses can provide a more comprehensive view of osteoporosis pathogenesis^77^, as such analysis is better equipped to link the drivers of a disease (e.g., genetic variations) with relevant functional outcomes (e.g., differential gene expression or metabolite abundance). Our data-intensive analysis framework, as an integrative approach, allows for the analysis of three or more data sets simultaneously to better advance our understanding of osteoporosis pathomechanisms, effects of calcium supplementation and fracture risk.

Osteoporosis is a systemic bone disease characterized by deterioration of the bone microstructure and decreased bone mass, which leads to increased risk of fractures and bone fragility^78^. An omnigenic model or effect is widely thought to be a reasonable way to explain and understand such complex diseases^23,24^, as such a model posits that small changes in hundreds or even thousands of genes can lead to a large outcome (i.e., disease). Indeed, such a model, where multiple genes or gene networks, instead of individual genes, can have a combinatorial effect may contribute to osteoporosis initiation, progression and treatment. Further, the biological network is not limited to just a gene network. Rather, an omnigenic effect could be reflected on different biological levels according to the genetic central dogma. The multi-modal model characterizing multi-omics information within different kinds of biological molecules (e.g., DNA, RNA or metabolites) can provide a more plausible scheme for an omnigenic effect than simply small changes in only gene expression. Here, our construction of the first multi-omics landscape of osteoporosis is a valuable data resource to help the field study such a complex disease, especially if one appreciates that an omnigenic effect in the form of a genotype-molecular phenotype association is related to osteoporosis indicators and treatment. With this aim in mind, we implemented a data-intensive analysis framework based on deep latent fusion analysis to extract the shared and specific molecular information for characterizing osteoporosis, in accordance with the principle of a data-intensive research paradigm.

We first confirmed the existing complementary biological information, which supported the relevance and novelty of using a multi-omics approach, rather than individual-omics approaches, for studying such a complex disease like osteoporosis. A recent study reported that the decreased bacterial richness and diversity in postmenopausal osteoporosis and metabolites were closely associated with such gut bacterial variation, where both serum procollagen PlNP and CTX-1 correlated positively with osteopenia-enriched *Allisonella*, *Klebsiella* and *Megasphaera*^79^. Our analysis similarly revealed that *Klebsiella*, *Intestinibacter*, *Streptococcus* and *Veillonella* are relevant to osteoporosis, as well as identifying their associations with metabolite signatures and how they complement BTMs.

Based on this analysis we identified two subtypes of osteoporosis and their corresponding M3S. The subtypes are distinguished by their associations with relevant osteoporosis phenotypes — notably, sensitive responders (CIS1) and non-sensitive responders (CIS2) within osteoporosis — and these subtypes are supported by follow-up outcomes. Especially as shown by the medical history, the clinical intervention should cause the improvement of osteoporosis, mainly because there were more osteoporosis individuals than others would take calcium supplements. Meanwhile, there were not significant difference between osteoporosis subtypes, indicating the difference of prognosis or intervention sensitivity between CIS1 and CIS2. Actually, the CIS1/CIS2 subtypes can be characterized from different perspectives: (1) at the phenotypic level, CIS1 and CIS2 show notable differences in several key osteoporosis indices, e.g., BMD, BTMs and response to calcium supplementation; (2) at the molecular level, CIS1 and CIS2 have significant differences in candidate signatures from different omics data, e.g., the epigenetic difference in the regulation of the calcium pathway; (3) in terms of fracture risk, CIS1 had a lower fracture rate compared to CIS2 according to the 4-year follow-up results. Whether they show significant disease progression or conversion is still an open question and will be of great use for future mechanical studies.

The xQTL model targeting M3S supplied quantitative association between genotype and these molecular phenotypes. The M3S xQTL model revealed many osteoporosis-related pathways and their functional representations on diverse molecular phenotypes, and especially supplied a complementary scheme (as sensitive and non-sensitive responders), or a Yin-Yang scheme, for the subtypes while explaining an omnigenic effect on osteoporosis. After narrowing down several conventional and new pathways associated with osteoporosis, we have a few insightful systematic observations. Notably, the two subtypes have molecular associations with well-known Wnt, Notch and RANKL signaling pathways. But individuals in the group of sensitive responders (i.e., CIS1, or the Yang-type subgroup) have relatively more M3S associations with AMPK-signaling pathway, which is of interest as AMPK signaling is a molecular coordinator of bone and energy metabolisms with an important role in skeletal physiology^80^. In contrast, individuals in the group of non-sensitive responders (i.e., CIS2, or the Yin-type subgroup) have relatively more M3S associations with the calcium signaling pathway and many different pathway members. Of note, individuals in the Yin-type subtype tended to have M3S associations with different types of molecular phenotypes, while individuals in the Yang-type subgroup have greater M3S associations with the microbiota and metabolites, suggesting more dysfunctional risk factors beyond host genetics. All these results strongly suggest the multi-modal characteristics of an omnigenic effect on osteoporosis, while highlighting the greater contribution of multi-omics rather than individual-omics signatures to identifying cases of sensitive responders for clinical intervention of osteoporosis.

By combining our genotype-molecular phenotype association study with additional public GWAS and EWAS data sets, we identified many co-occurrences between osteoporosis and other chronic diseases, including chronic obstructive pulmonary disease (COPD), Alzheimer’s disease and diabetes. Indeed, patients with COPD have a greater risk of bone fractures and osteoporosis, and use of high-dose inhaled corticosteroids increase this risk further, including for four of the specific osteoporosis-related events: all fractures, fractures typically related to osteoporosis, prescriptions of drugs for osteoporosis and diagnosis of osteoporosis^81^. And Alzheimer’s disease has a higher incidence in older women, with a spike in cognitive decline that tracks with increased visceral adiposity, dysregulated energy homeostasis and bone loss during the menopausal transition. There is a causal role for rising serum follicle-stimulating hormone (FSH) levels in the exaggerated Alzheimer’s disease pathophysiology during menopause, indicating an opportunity for treating Alzheimer’s disease, obesity and osteoporosis with a single agent (notably, a neutralizing anti-FSH antibody^82^). These associations supported recent robust association studies about risk factors for common diseases; e.g., screening genotypes for common variants that affect the risk of osteoporosis and other diseases, whose information will affect decisions made by individuals with regard to their health care^23^.

Furthermore, we built the osteoporosis risk model based on M3S, and it outperformed baseline models based on conventional risk covariates and BTMs, providing new candidates for osteoporosis diagnosis, prognosis and early prevention. BTMs can be used to study changes in bone remodeling in osteoporosis, which can provide information that is useful for the management of patients with osteoporosis, for both the initial clinical assessment and for guiding and monitoring of treatment. However, investigators and clinicians should be aware of the appropriate sample collection and storage conditions for optimum measurements of these markers^83^. Thus, identifying markers based on diverse molecular phenotypes would provide new candidates to easily and accurately diagnose or prognose osteoporosis. Research has shown that abnormal epigenetic regulation could cause dysregulation of bone metabolism and lead to osteoporosis through its important roles in osteogenic differentiation and the pathogenesis of osteoporosis^47^. Of note, the presence of a “microbiota-skeletal” axis would be explained by the influence of the gut microbiota on skeletal homeostasis via effects on host metabolism, immune function, hormone secretion and the gut-brain axis^84^. Thus, in our multi-omics landscape of osteoporosis, the epigenetics, metagenomics information and their regulated metabolomics information combined provides a complete and complementary resource for investigating the pathogenic mechanisms of osteoporosis, while recognizing the utility of M3S in addition to conventional BTMs. With the functional relevance and osteoporosis associations of M3S, the risk or diagnosis model is efficiently learned with improved discrimination on osteoporosis and its subtypes. Indeed, the complete genotype-phenotype viewpoint of osteoporosis is necessary and important to understand the underlying molecular mechanism of the disease and to identify new subtypes. Meanwhile, in predictive applications, microbiota and metabolite modals are robust for osteoporosis identification, and all three modals are efficient for osteoporosis subtype recognition. Thus, the diagnosis and prognosis models apply whole or part candidate components or biomarkers from M3S, which can provide flexible test panels to clinical applications when only certain modal signatures are available; e.g., testing with single modality from incomplete multi-modal learning^85^. For early diagnostic standards such as osteoporosis diagnosis or fracture risk assessment, multi-omics signatures and models are efficient, as shown in this work, however they would require huge human omics data, potentially requiring remarkable human and material resources. In fact, as our experiments show, DNA methylation data have made a remarkable contribution to osteoporosis diagnosis and fracture risk assessment. Thus, by further developing the deep learning model, it is possible to achieve similar clinical application results by using only DNA methylation data or other monomodal data.

Finally, our data and analyses also demonstrate the existence of an osteoporosis-specific fracture risk and its potential biomarkers and provide new evidence and analytical support for precision medicine in osteoporosis. Of note, we are currently analyzing fracture outcomes over a 4-year period, whereas traditional clinical trials on fractures would require follow-up for more than 20 years^86^. Therefore, it is expected that, our data and analyzes will contribute to conducting more diverse and personalized assessment of fracture risk as we obtain longer-term follow-up fracture results from our study cohort in the future.

Notably, the osteoporosis and its subtypes characterized by M3S can represent an anchor for future studies of the pre-disease state^87^ of osteoporosis. The early-warning signal^88^ corresponding to the development and progression of osteoporosis should be associated with M3S, indicating the temporal/causal functional cascade of bone remodeling imbalance. In the clinic, we can expect to continue developing an individual risk model^89^ that predicts the likelihood probability of developing osteoporosis in people with osteopenia and fracture in people with osteoporosis.

Collectively, our analyses of the multi-omics landscape of osteoporosis in Chinese populations have identified new M3S for osteoporosis and its two subtypes. On one hand, many snpGenes are associated with these signatures and reveal diverse candidate biological mechanisms underlying osteoporosis, especially the xQTL preferences of osteoporosis and its subtypes which are in the form of pleiotropism or physiological associations on different biological levels/domains. And subtypes are also observed different sensitivities to calcium supplementation, which could explain the heterogeneity in efficacy of calcium supplementation and better guide osteoporosis medication. On the other hand, M3S can be thought of as functional representations of hidden genotypes, and they are the basis of improved osteoporosis and fracture risk models in addition to conventional risk factors and BTMs. All these integrative data resources and explainable data-intensive analysis framework should greatly help future studies into the early diagnosis of, and personalized treatment for, osteoporosis and any other co-occurring complex diseases.

## Materials and methods

### Recruitment of the participants

The study was a part of the registered protocol at Clinicaltrials.gov (NCT02958020), and the protocol was approved by the Institutional Review Board at Longhua Hospital affiliated to the Shanghai University of Traditional Chinese Medicine (2016LCSY065). It was performed in accordance with the ethical standards laid down in the 1964 Declaration of Helsinki and its later amendments or comparable ethical standards. Residents from the Lujiazui and Jinshan subdistricts in Shanghai, China, participated in a multicentered prospective study from September 2019 to November 2023, and all participants have provided informed written consent before participation. The detailed criteria for participation can be found in our previous reports about the CCCO cohort^90^.

### Questionnaires

All participants have completed paper-based questionnaires via face-to-face interviews, and the questionnaires contained information about education level, smoking status, province, age, gender, etc. Based on the outpatient and emergency treatment record books provided by the participants, information about medication history of calcium supplements, vitamin D supplements and bisphosphonates was also collected.

### Physical examinations

All participants were measured barefoot for height and for body weight while wearing indoor clothing and their body mass index (BMI) was calculated as weight (kg)/height squared (m^2^).

### Measurement of serum bone metabolism markers and calcium and phosphorus metabolism indicators

The venous blood samples of all participants were collected after an overnight fasting in non-EDTA-containing tubes. These blood samples were centrifuged within 2 h after the blood collection at 3000 rpm for 15 min at room temperature to separate the serum. The electrochemiluminescence immunoassay was applied to measure serum concentrations of N-terminal propeptide of osteocalcin (OST), β-C-terminal telopeptide of type I collagen (β-CTX), and type I collagen (PINP). The continuous monitoring technique was used to measure serum alkaline phosphatase (ALP) concentration. The sensitive and specific high-performance liquid chromatography–tandem mass spectrometry (HPLC–MS/MS) method detected total 25(OH)D (25(OH)D3 and 25(OH)D2). The chemiluminescence immunoassay detected parathyroid hormone (PTH). The o-cresolphthalein-complexone method and phosphomolybdate ultraviolet colorimetry were used to measure total calcium (Ca) and phosphorus (P), respectively.

### Assessment of BMD, osteoporosis diagnosis and fracture definition

The dual-energy X-ray absorptiometry densitometer (DXA, Hologic Discovery CI, Bedford, MA, USA) instruments were applied to measure the BMD values of each single lumbar vertebra (L1–L4), the total lumbar vertebra (L1–L4) and the total left hip joint (including femoral neck). All the centers used the same model of instruments, which passed the annual verification, and daily calibration program was performed each time the instrument was powered on.

For osteoporosis diagnosis, the BMD values were expressed as T-scores (number of standard deviations above/below the mean peak BMD of healthy young-adults of the same race and same gender). Participants with T-scores of any site ≥ −1.0 were considered not having osteoporosis or osteopenia; participants with T-scores of less than −1.0, and more than −2.5 were thought as having osteopenia; and those with T-score ≤ −2.5 were diagnosed as having osteoporosis, which are in accordance with criteria of the World Health Organization and the guideline for diagnosis and treatment of primary osteoporosis issued by the Chinese Society of Osteoporosis and Bone Mineral Research in 2017.

To maximize statistical effectiveness, we have employed a well-known inclusive definition of fractures^74^. Cases were included that had fractures confirmed at any skeletal site by medical, radiological, or questionnaire survey reports. However, finger, toe and skull fractures as well as fractures with high trauma were excluded when possible.

### Statistical analysis

The normality of the demographic and clinical data was assessed by the Kolmogorov–Smirnov test. The normally distributed variables were expressed as mean ± std, and the skewed distributed variables were expressed as median (interquartile range). The ANOVA was used to compare the differences among groups with normally distributed data, and the Kruskal–Wallis test was used to compare the differences among groups with nonnormally distributed data.

All statistical analyses were performed in the SPSS software (version 26.0, SPSS Inc. of IBM, USA), with a *P* < 0.05 considered statistically significant.

### Genomics data and GWAS calculation

Genotyping of 412 samples was performed using the Illumina Infinium Global Screening Array that analyzes over 710,000 SNPs. It is a fully custom array designed by WeGene (https://www.wegene.com/). SNPs were excluded if they had a call rate < 95%, MAF < 5% and *P* value of violations from Hardy–Weinberg equilibrium (PHWE) < 0.00001. After quality control, 691,352 SNPs of 412 samples were left for further analysis. Imputation was done by SHAPEIT2 and IMPUTE2 using 1000 genome phase 3 as reference. Imputed variants were filtered with MAF > 0.01, imputation quality score > 0.6 and violations from Hardy–Weinberg (HW) equilibrium (*P* < 1 × 10^−5^). We further removed four samples to ensure any relativeness up to the second degree in all the sample pairs (www.cog-genomics.org/plink/1.9/)^91^, leaving 8,324,631 variants of 408 samples (322 females and 86 males). All GWASs were conducted by PLINK2 (www.cog-genomics.org/plink/2.0/)^92^. GWASs were performed by a linear regression and additive model adjusted for age, sex and top 8 PCs.

### Methylation data and EWAS calculation

Bisulfite conversion of five hundred nanogram of genomic DNA from each whole blood sample was performed using the EZ DNA Methylation Kit (Zymo Research, Irvine, CA). Genome-wide DNA methylation was profiled on Infinium Human Methylation 850 K EPIC BeadChip (Illumina, San Diego, CA) following the manufacturer’s instructions. Samples were randomized with respect to slide and position on arrays, and all samples were hybridized and scanned concurrently to mitigate batch effects as recommended by the Illumina Infinium® HD Assay Methylation Protocol Guide. Illumina .idat files were then processed with the ChAMP Bioconductor package^93^ without background correction. Probes with SNPs and on chromosomes X and Y were removed. Normalization and quality control was conducted by applying BMIQ^94^ and champ.QC incorporated in ChAMP. Based on champ.SVD, we found a significant slide/beadchip effect. Therefore we used ComBat^95^ incorporated in ChAMP on *M*-values (logit of beta-values) to correct for the slide effect and then transformed the M-values back to beta-values. Finally, there were 413 samples and 713,195 CpGs left for further analysis. All the EWASs were conducted by using R package limma^96^, and adjusted for age, sex, BMI, cell composition, the top 10 PCs, smoking history and drinking history.

### Metabolite data and mass spectrometry analysis

To diminish sample degradation, samples were thawed in an ice-bath, and 20 μL of plasma was added to a 96-well plate, which was transferred to the Eppendorf epMotion Workstation (Eppendorf Inc., Hamburg, Germany) and was finally sealed for LC-MS analysis. All targeted metabolites were quantified by an ultra-performance liquid chromatography coupled to tandem mass spectrometry (UPLC-MS/MS) system (ACQUITY UPLC-Xevo TQ-S, Waters Corp., Milford, MA, USA) in a well-established pipeline with quality control^97^. The raw data files generated by UPLC-MS/MS were processed to perform peak integration, calibration and quantitation for each metabolite by the MassLynx software (v4.1, Waters, Milford, MA, USA), where the obtained clean data can be applied for downstream analysis^98^.

### Gut microbiome data and metagenomics analysis

All the enrolled participants were provided with a stool collection kit. Each participant collected a bowel movement using a disposable plastic bedpan covered with toilet paper. Participants were instructed to collect a sample of specimen with a plastic applicator attached to the cap and to place the applicator into a tube with stabilizer (Stool preservation liquid, Realbio Technology), and then shake the tube to mix stool and preservative. Samples were carried to the physical examination center within 24 h, and were kept in a portable Styrofoam box with dry ice and shipped to the laboratories at Shanghai Sinomics Corporation (Shanghai, China) within 6 h and stored in a −80 °C freezer until the nucleic acid extraction. DNA was extracted using Qiagen QIAamp DNA Stool Mini Kit (Qiagen) following the standard protocol. The extracted DNA was prepared by KAPA Hyper Prep Kit (Illumina) for whole-genome sequencing. A paired-end (2 × 150 bp reads) shallow shotgun metagenomic sequencing was carried out using an Illumina NovaSeq 6000 platform (Illumina, USA) at Shanghai Sinomics Corporation (Shanghai, China).

Taxonomic and functional profiles were analyzed using the bioBakery workflow^99^. Raw sequencing reads were performed for quality control by the KneadData pipeline with default parameters to filter out low-quality and human-origin reads^99^. After quality control, the average reads per sample was 9.8 million (range 5.7 million–20.0 million). Taxonomic features were profiled using MetaPhlAn^99^. Functional potential was profiled using the HUMAnN 3^99^ and UniRef90 database^100^.

### Differential analysis of individual-omics data

PCA was used to visualize the data distribution of samples in different groups, and partial least squares discriminant analysis (PLSDA) was used to observe the distinguishing data distribution of sample groups. For each type of omics data, the differentially expressed features/molecules (e.g., DEM including genes or methylation sites) were initially selected and summarized by Wilcoxon test and significance threshold is *P* < 0.05. The co-expression profiles among features were calculated by spearman correlation for each type of omics data in each sample group. WGCNA^29^ was applied for co-expression module analysis. RV index^30^ was used to estimate the information overlapping or correlation among different omics datasets.

### Integrative osteoporosis subtype identification by deep latent space fusion model

For integrative analysis of multi-omics data, the matched 366 samples were determined, including from 91 normal individuals, 158 individuals with osteopenia and 117 individuals with osteoporosis. Their corresponding methylation, metabolite and microbiota data were used as input for our DLSF pipeline^16^. On these data, three baseline methods were also calculated and compared, including PFA^17^, SNF^31^, and PINS^32^. The parameters of different methods were adopted in a series of values by grid strategy in the suggested ranges, and potential number of sample clusters was set from 2 to 4. For each calculation outcome, the sample cluster was evaluated by the sample balance between different clusters, and their associations with different clinical phenotypes, where significance test used Wilcoxon test and threshold is *P* < 0.05.

Of note, another set of samples from a Jinshan cohort belonging to CCCO was collected in the year 2021 for independent testing. In this new cohort, there were 157 samples with existing 16S data, and 162 samples with targeted CpGs and metabolites according to M3S from our above analysis. They could be used for individual-omics testing, and the matched 87 samples (53 normal and 34 osteoporosis samples) were applied for multi-omics test (e.g., risk model assessment).

### Downstream multi-omics analysis for osteoporosis and its subtypes

For the downstream analysis of different omics signatures, Wilcoxon test was used for significance testing of differential expression analysis; the heatmap was used to show the hierarchical structure of signatures and their expression pattern among different osteoporosis subtypes; association matrix was used to illustrate the co-expression relationship among different modal signatures and phenotypes; stemness-like index approach^101^ was used to select high-weights multi-modal molecular features as M3S; functional enrichment was applied to analyze the functional significance of a group of genes or metabolites^102^; EWASdb was used to annotate the phenotype relevant to methylation signatures^49^; metabolic enzymes (level 4 Enzyme Commission [EC] nomenclature) and complete metabolic pathway from MetaCyc^103^ in the community and per organism, were quantified for bridge the functional relevance with microbiota and metabolite.

In addition, to investigate the genotype characteristics corresponding to the osteoporosis subtype determined by omics signatures, two kinds of snpGenes were identified, i.e., genes where annotated SNPs located or neighbored according to ANNOVAR^104^. On one hand, the SNPs whose value distribution has significant change among different sample groups based on *χ*^2^ Test were filtered and their annotated genes were collected as global snpGenes. On the other hand, the targeted xQTL model was carried out, including methylationQTL, metaboliteQTL, and microbiotaQTL, by using Plink2. The SNPs involved in different QTLs were filtered by *P* < 10^−5^ and their annotated genes were collected as xQTL or local snpGenes. The STRING database^105^ was used to extract protein association network among snpGenes. The Comparative Toxicogenomics Database^63^ was applied to extract the osteoporosis-relevant inference networks associated to snpGenes. The clusterprofiler^106^ was carried out for functional enrichment analysis of snpGenes.

Logistic regression (LR) model was learned based on different features, i.e., conventional risk factors, BTMs, and M3S. Of note, the microbiota signatures of M3S used features on the genus level because only 16S data were available as independent data from the Jinshan cohort. A decision curve was used to evaluate the clinical utility of discussed LR models. ROC curve (receiver operating characteristic curve) and AUC (area under curve) were used to evaluate the prediction performance of LR models. For performance assessment of each model, 70% of the samples from the CCCO cohort were used as a training dataset, and the other 30% of the samples were used as a test dataset, which determined an optimal M3S subset through mRMR^107^. The final risk model was built with the CCCO cohort based on such optimal M3S subset, and tested on all independent samples from the Jinshan cohort. The risk models were obtained in a similar way for osteoporosis identification and osteoporosis subtype recognition, respectively. The optimal M3S subset for osteoporosis identification included Klebsiella, Veillonella, Intestinibacter, 9E-tetradecenoic acid, and Suberic acid. The optimal M3S subset for osteoporosis subtype recognition included cg22026953, cg18008345, cg21813369, cg11070274, cg00329101, cg23088318, cg15508935, cg09656629, cg15320980, cg00431894, cg18243574, Klebsiella, Streptococcus, Veillonella, Threonic acid, and 5Z-Dodecenoic acid.

The ranking assessment was used to evaluate the predictive power of different features on fracture risk, and the AUC was the quantitative measurement. The AUC is between 0 and 1. The larger the AUC, the more effective the fracture prediction. In addition to the individual features, a simple composite index based on the sum of member features (*N* < 5) was also calculated and evaluated by AUC, which helps to obtain a more accurate fracture prediction. Focusing on methylation, a small panel of four CpGs (i.e., cg15320980, cg18249968, cg15508935 and cg23088318) can provide an effective composite index to assess fracture risk.

### Supplementary information

Supplementary Figures 1-24

Supplementary Tables 1-17

## Data Availability

The genomics, epigenomics and metagenomics data can be viewed in NODE (http://www.biosino.org/node) by entering the accession (OEP003842) into the text search box or through the URL: http://www.biosino.org/node/project/detail/OEP003842. The xQTL summary data can also be accessed from OEP003842.
