# Supplementary material for: Multi-modal molecular determinants of clinically relevant osteoporosis subtypes
Source: Cell Discov. 2024 Mar 12;10:28. doi: 10.1038/s41421-024-00652-5 (PMC10933295; doi:10.1038/s41421-024-00652-5)

Suppl. Figure 1 - Cluster balance evaluation for DLSF and other baseline methods.

The comparison between DLSF and other multi-omics methods is measured using the balance index, the largest value of which is expected to indicate a similar number of samples in different clusters/subtypes to avoid the noise from extremely small clusters.

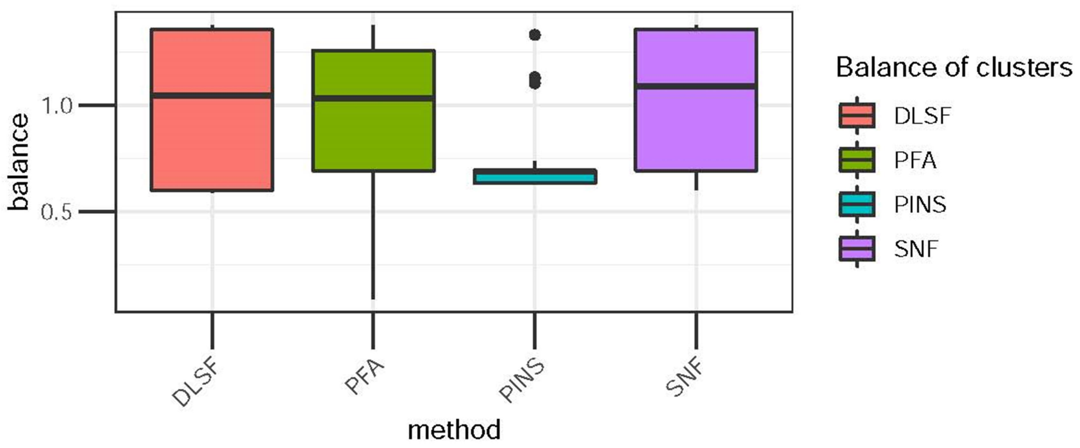

Suppl. Figure 2 - Clinical phenotype associations for osteoporosis subtypes identified by DLSF.

The associations between different clinical phenotypes and osteoporosis subtypes identified by the DLSF are estimated, and they indicate the possible clinical importance and significant phenotypic changes between different molecularly defined subtypes.

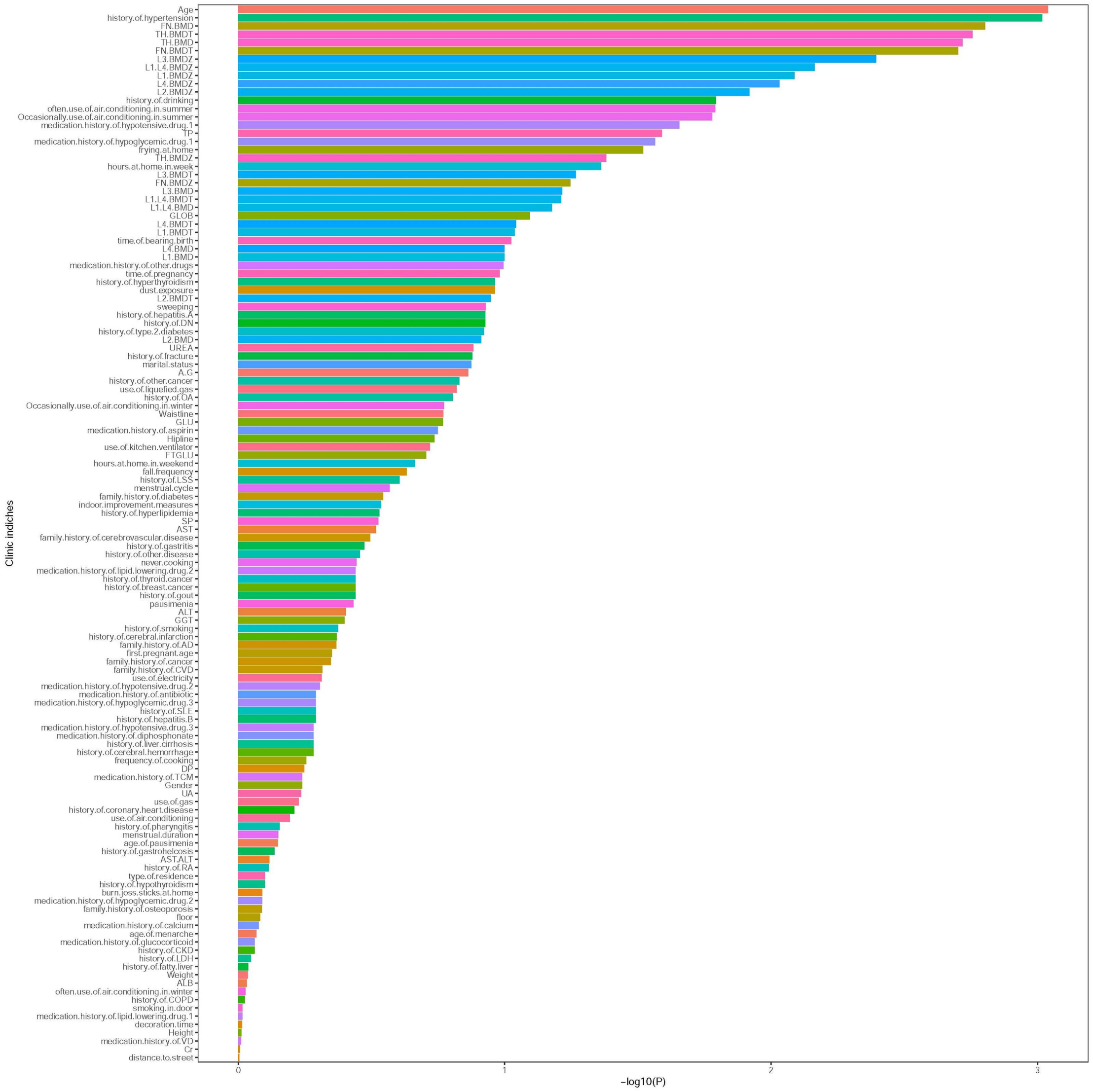

Suppl. Figure 3 - Clinical phenotype change between osteoporosis subtypes and normal.

The box-plot displays the value distributions of each phenotype index between two osteoporosis subtypes and normal groups.

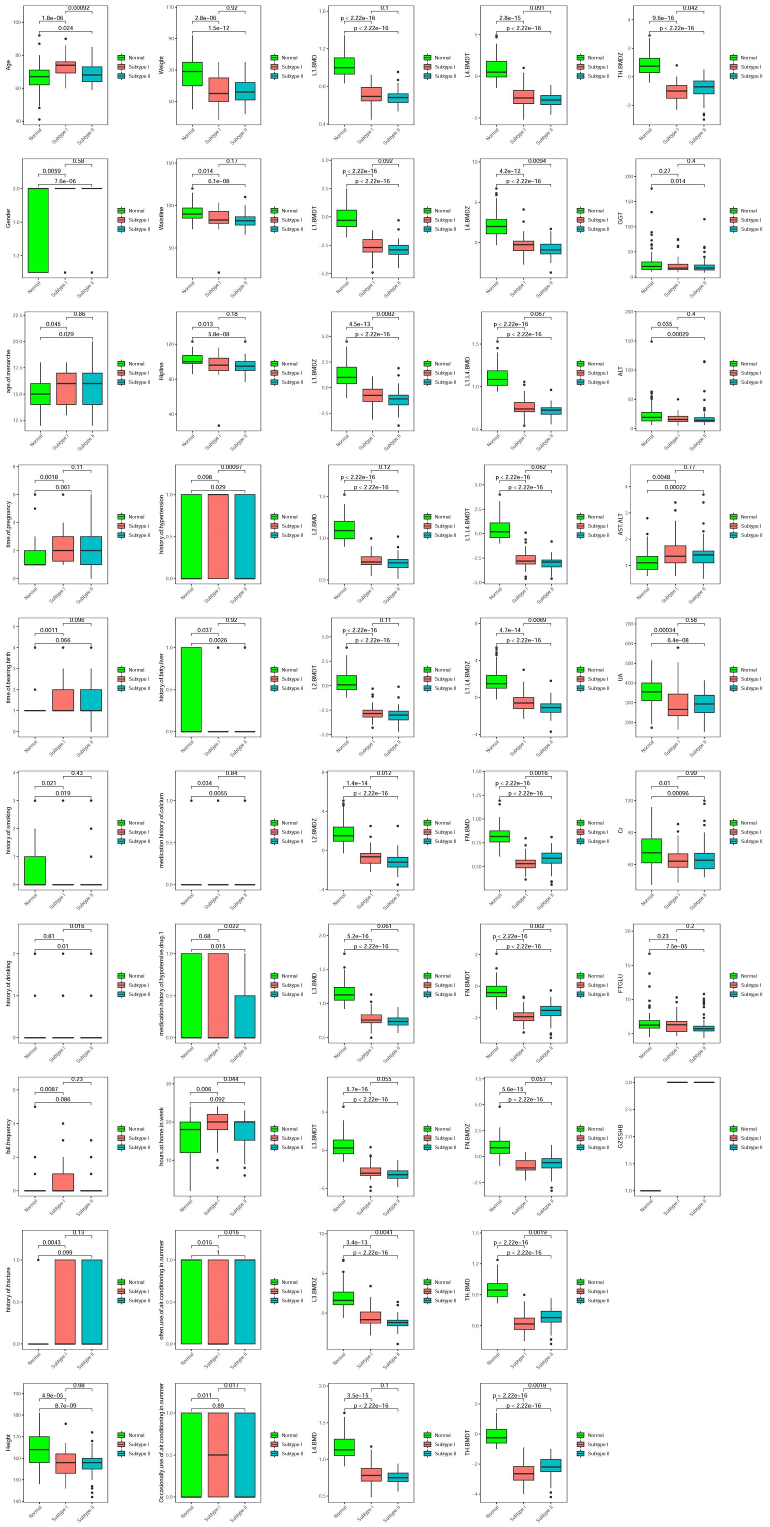

Suppl. Figure 4 – Survey of medical history for osteoporosis and subtypes.

The bar-plot indicates the percentage of individuals receiving particular medical intervention (e.g. vitamin D supplements or bisphosphonates), and their distribution differences between osteoporosis and others, or between two osteoporosis subtypes.

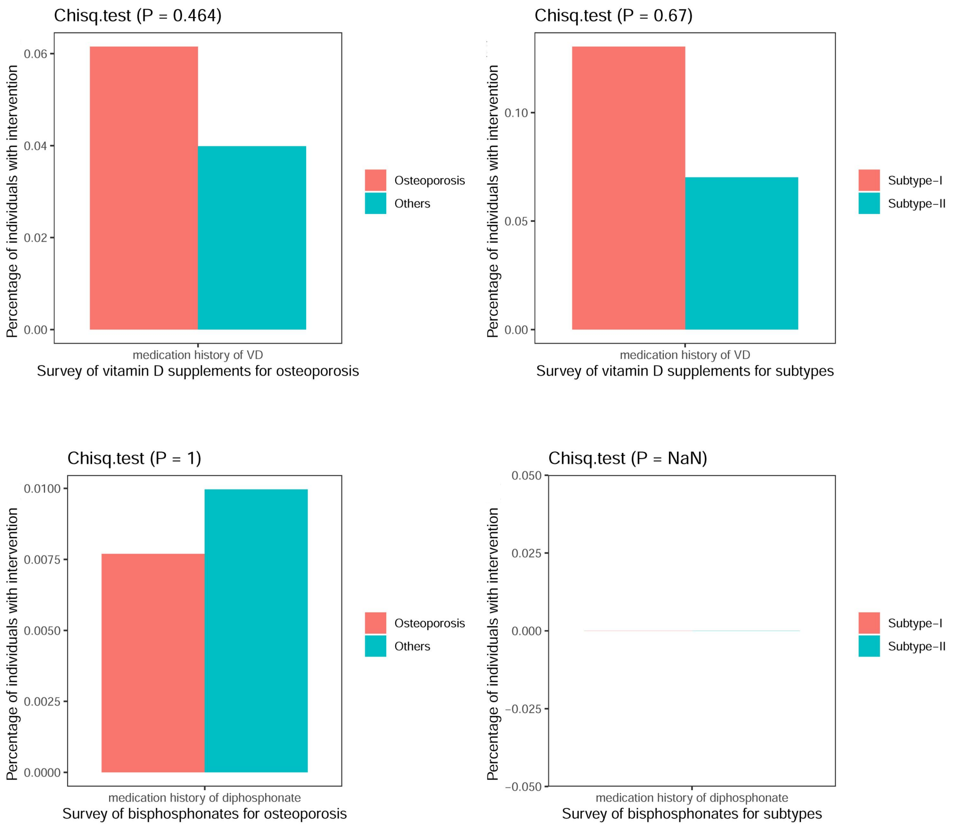

Suppl. Figure 5 - Differential expression pattern of M3S.

The box-plot displays the value distributions of each molecular marker in M3S between two osteoporosis subtypes, osteopenic and normal groups.

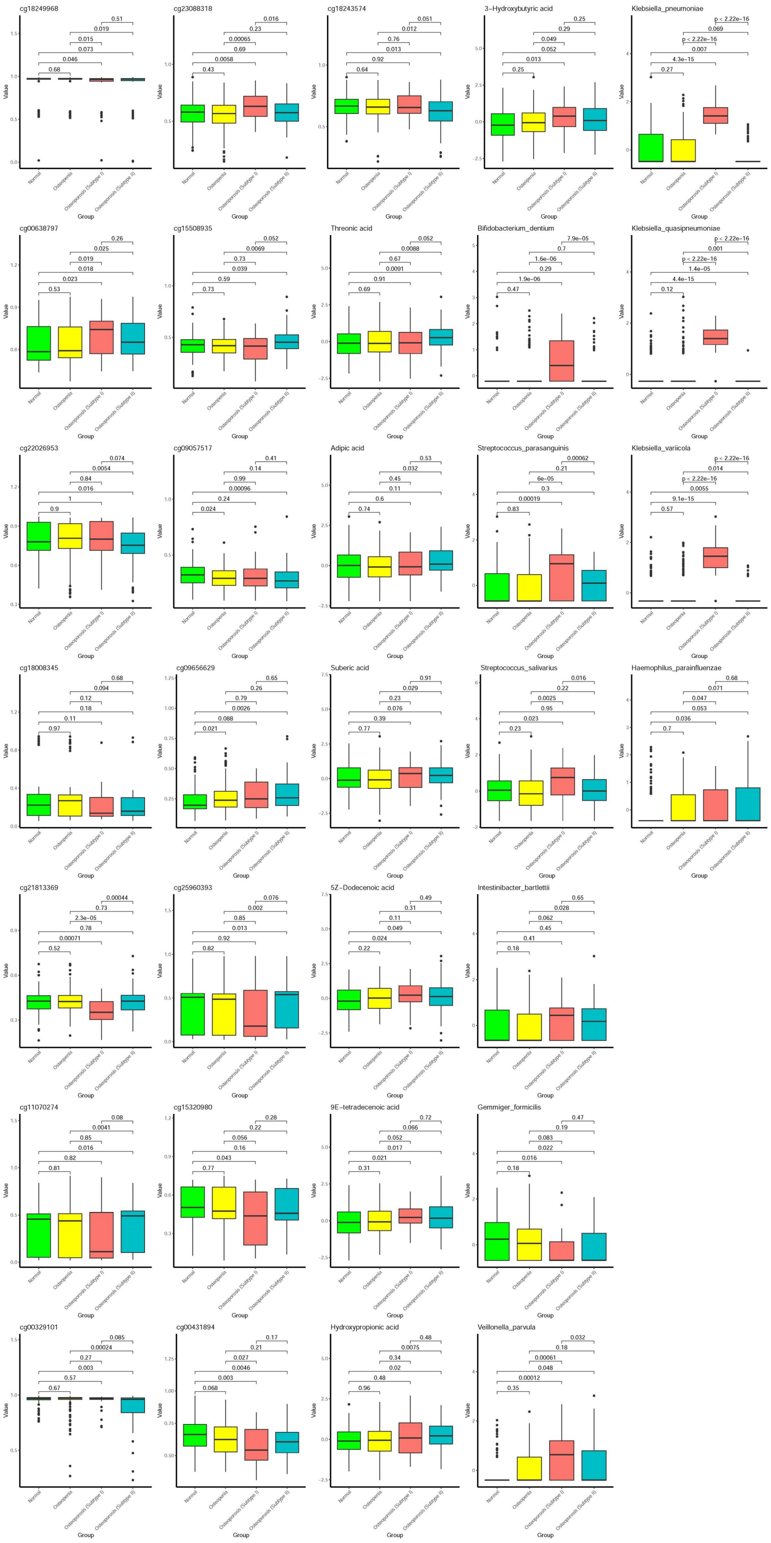

Suppl. Figure 6 - Differential expression pattern of microbiota component of M3S on genus level

The box-plot displays the value distributions of each microbiota marker in M3S between two osteoporosis subtypes, osteopenic and normal groups, whose values are on genus level.

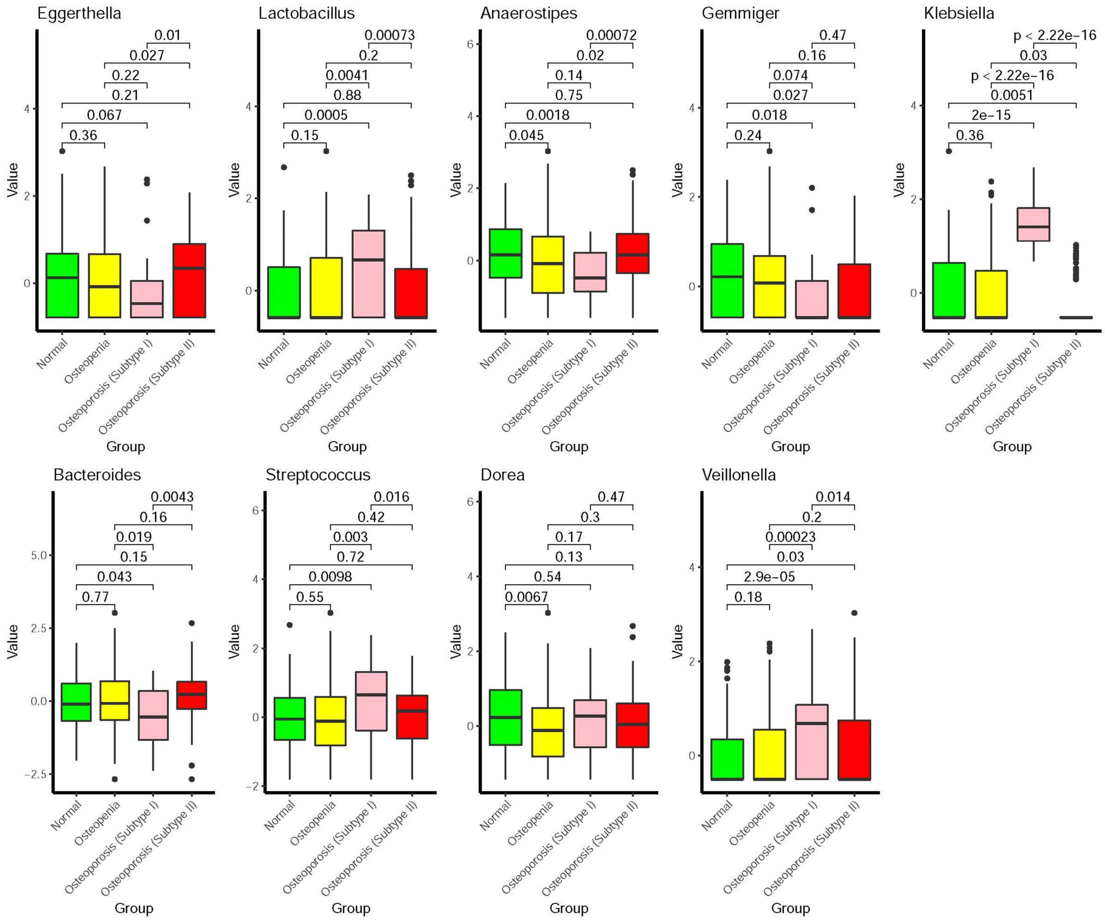

Suppl. Figure 7 - EWAS public reported phenotypes associated to methylation signatures in M3S

The heatmap summarizes the methylation signatures in M3S reported in the public EWAS database, where each column indicates a methylation signature; each row indicates a phenotype; and each matrix element in the heatmap indicates the number of records in the EWAS database with reports of the association pair between a particular methylation signature and a phenotype.

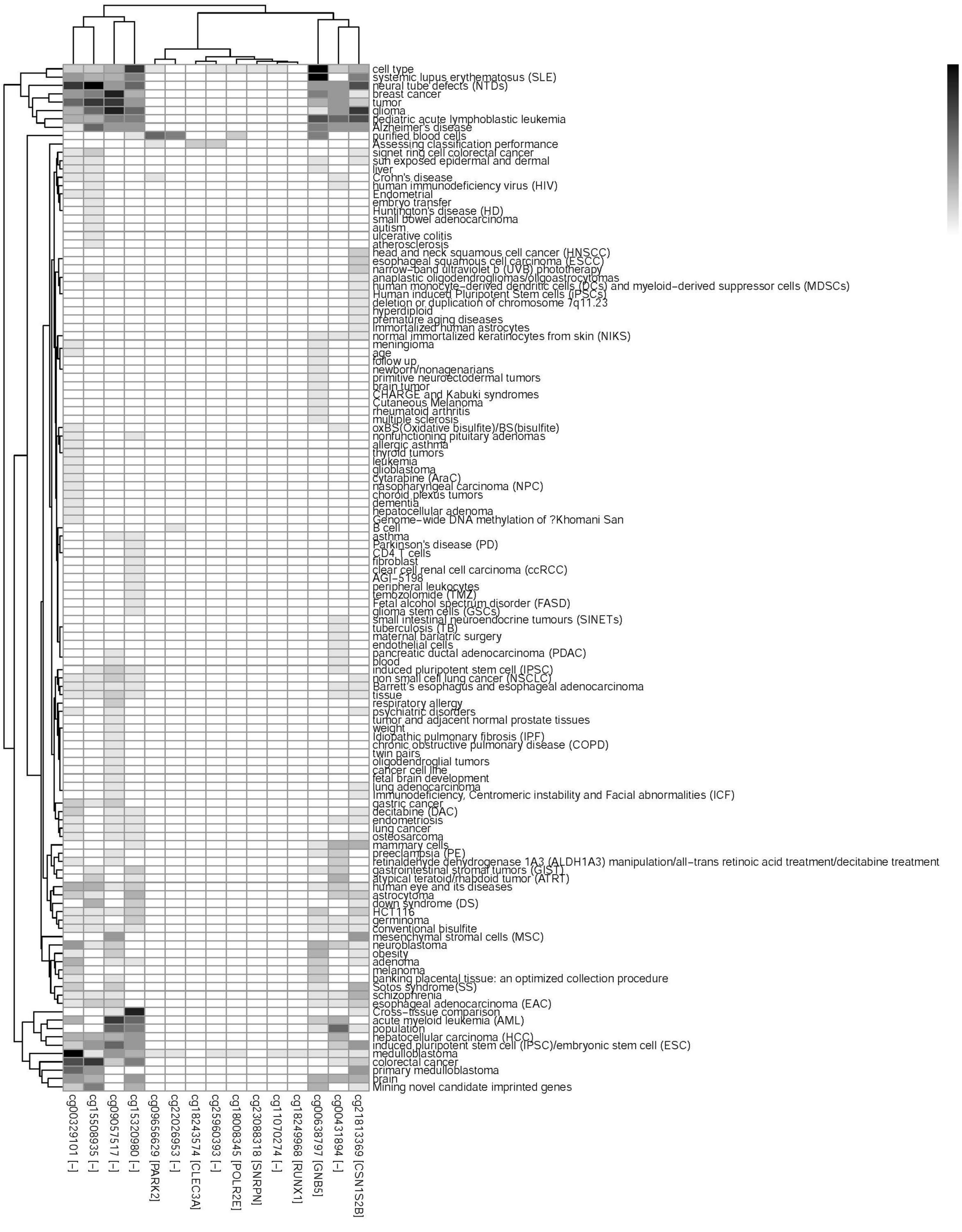

Suppl. Figure 8 - Mutation distribution pattern of global snpGenes.

The heatmap summarizes the frequency of SNVs observed in two osteoporosis subtypes, osteopenic and normal groups.

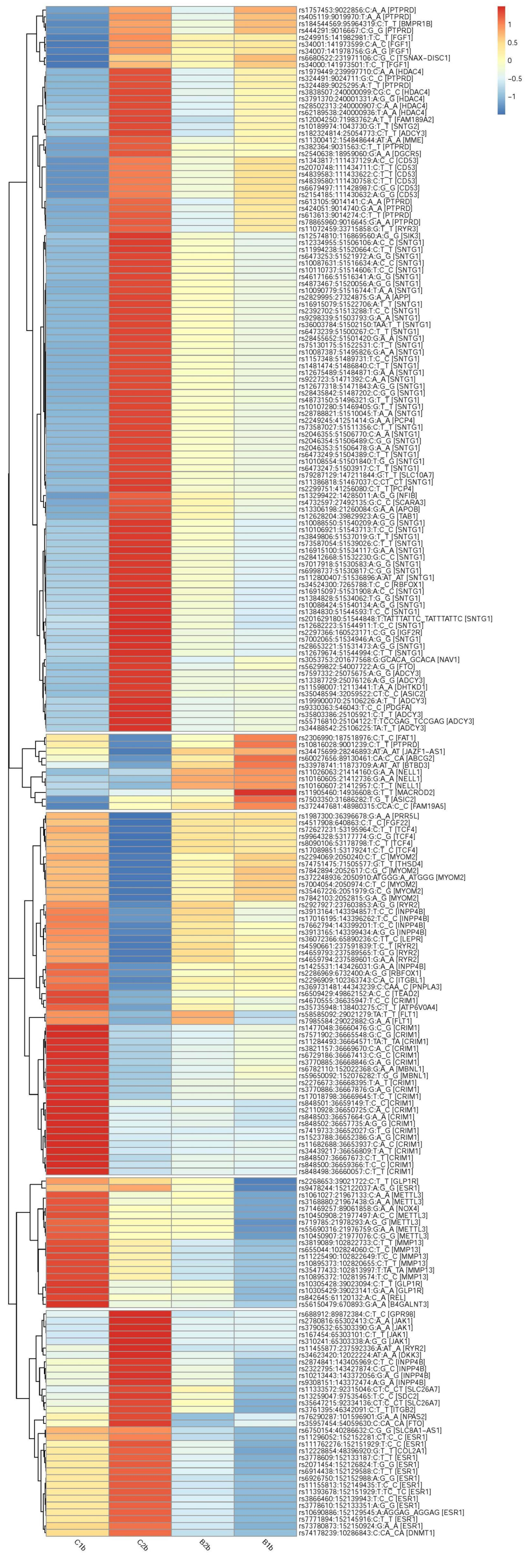

Suppl. Figure 9 - Overlapping statistic of global snpGenes and CDT genes.

The statistics of the overlap between snpGenes identified here and disease-associated genes reported in the CDT.

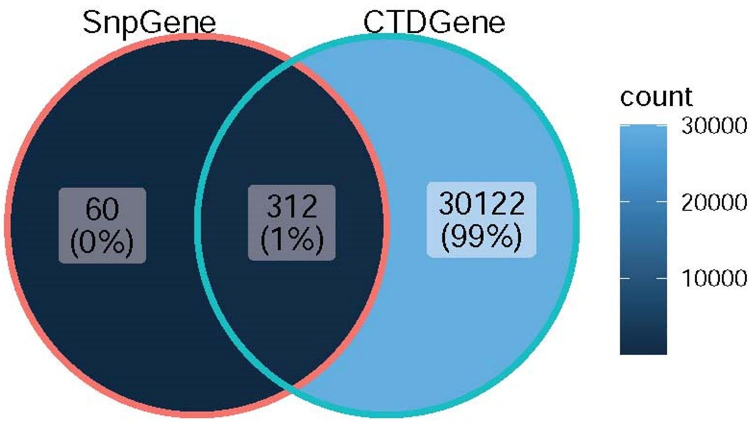

Suppl. Figure 10 - Functional enrichments of xQTL snpGenes.

Functional enrichments of snpGenes from subtype-specific xQTLs.

id	source	term_id	term_name	term_size		p_value
1	KEGG	KEGG:04360	Axon guidance	181		1.3e−02
2	REAC	REAC:R−HSA−112316	Neuronal System	400		5.5e−06
3	REAC	REAC:R−HSA−6794362	Protein−protein interactions at synapses	86		9.5e−03
4	REAC	REAC:R−HSA−392154	Nitric oxide stimulates guanylate cyclase	21		1.0e−02
5	REAC	REAC:R−HSA−1489509	DAG and IP3 signaling	41		2.7e−02
6	REAC	REAC:R−HSA−5173105	O−linked glycosylation	108		3.3e−02
7	REAC	REAC:R−HSA−112040	G−protein mediated events	54		5.0e−02

CIS1

id	source	term_id	term_name	term_size		p_value
1	KEGG	KEGG:04724	Glutamatergic synapse	114		1.4e−10
2	KEGG	KEGG:04713	Circadian entrainment	97		1.1e−06
3	KEGG	KEGG:04020	Calcium signaling pathway	239		1.1e−05
4	KEGG	KEGG:04921	Oxytocin signaling pathway	154		3.7e−05
5	KEGG	KEGG:04730	Long−term depression	59		4.9e−05
6	REAC	REAC:R−HSA−112043	PLC beta mediated events	49		4.7e−07
7	REAC	REAC:R−HSA−112040	G−protein mediated events	54		3.2e−06
8	REAC	REAC:R−HSA−1489509	DAG and IP3 signaling	41		9.0e−06

CIS2

Suppl. Figure 11 – Global methylation difference between CIS1 and CIS2.

Globally, CIS2 tends to have hyper-methylation compared to CIS1, indicating the potential gene expression suppression (i.e. expression down-regulation or pathway inhibition related to Calcium) occurred in CIS2.

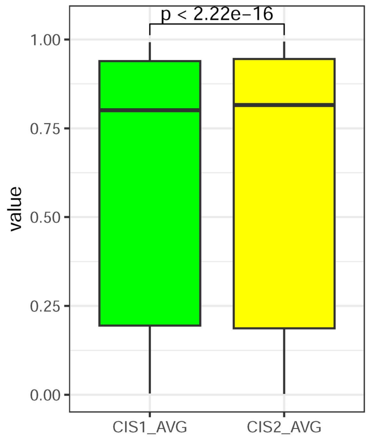

Suppl. Figure 12 - Local methylation difference between CIS1 and CIS2.

The cases of dys-methylation observed on different CpGs of several genes relevant to Calcium. Consistent to global pattern, many CpGs actually are in local high-methylation states in CIS2 compared to CIS1.

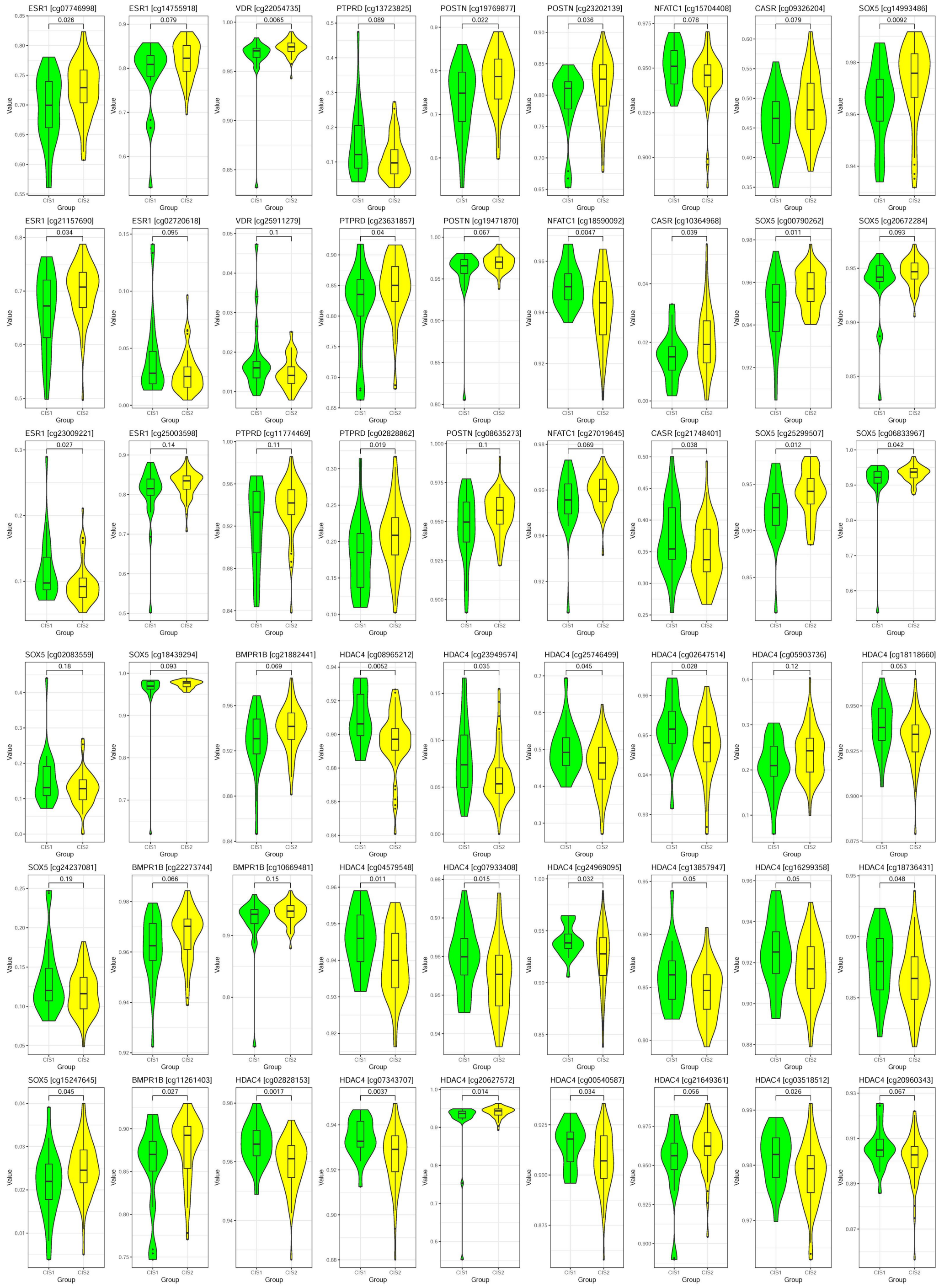

Suppl. Figure 13 - Validation of methylation-phenotype associations between CCCO and Jinshan cohorts.

The comparison of methylation-phenotype associations between CCCO and Jinshan cohorts is measured by the correlation matrix, where the lower triangular matrix indicates the methylation-phenotype associations observed in CCCO cohort and the upper triangular matrix indicate such associations observed in Jinshan cohort.

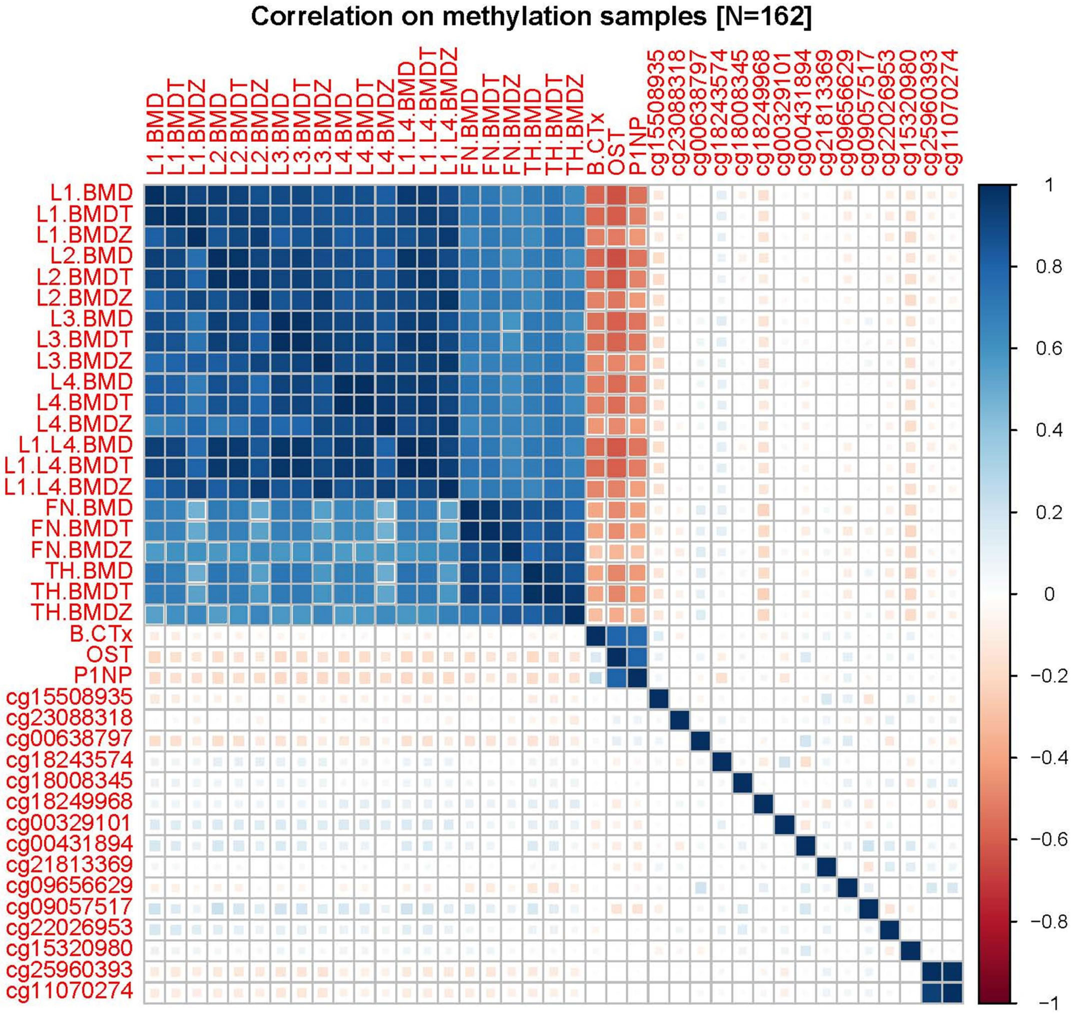

Suppl. Figure 14 - Validation of metabolite-phenotype associations between CCCO and Jinshan cohorts.

The comparison of metabolite-phenotype associations between CCCO and Jinshan cohorts is measured by the correlation matrix, where the lower triangular matrix indicates the metabolite-phenotype associations observed in CCCO cohort and the upper triangular matrix indicate such associations observed in Jinshan cohort.

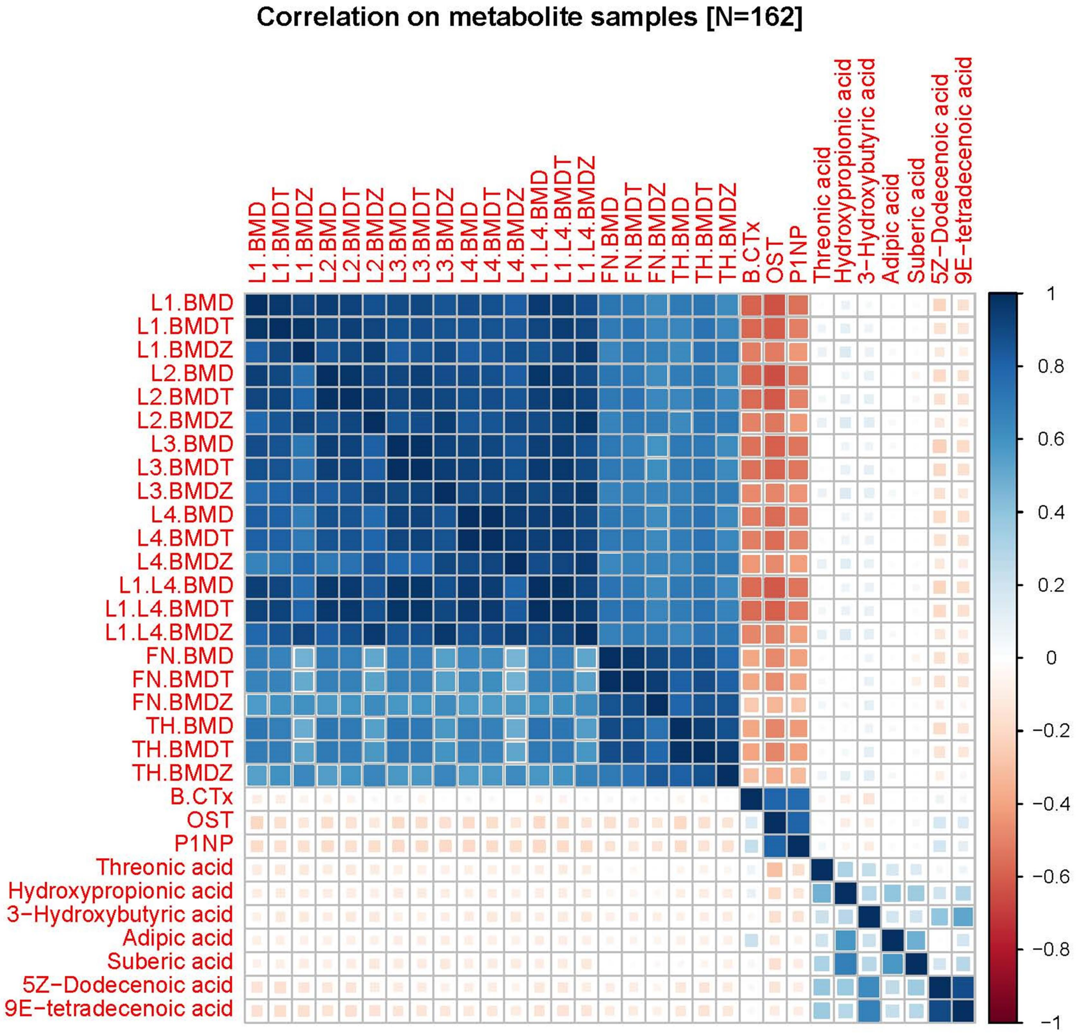

Suppl. Figure 15 - Validation of microbe-phenotype associations between CCCO and Jinshan cohorts.

The comparison of microbe-phenotype associations between CCCO and Jinshan cohorts is measured by the correlation matrix, where the lower triangular matrix indicates the microbe-phenotype associations observed in CCCO cohort and the upper triangular matrix indicate such associations observed in Jinshan cohort.

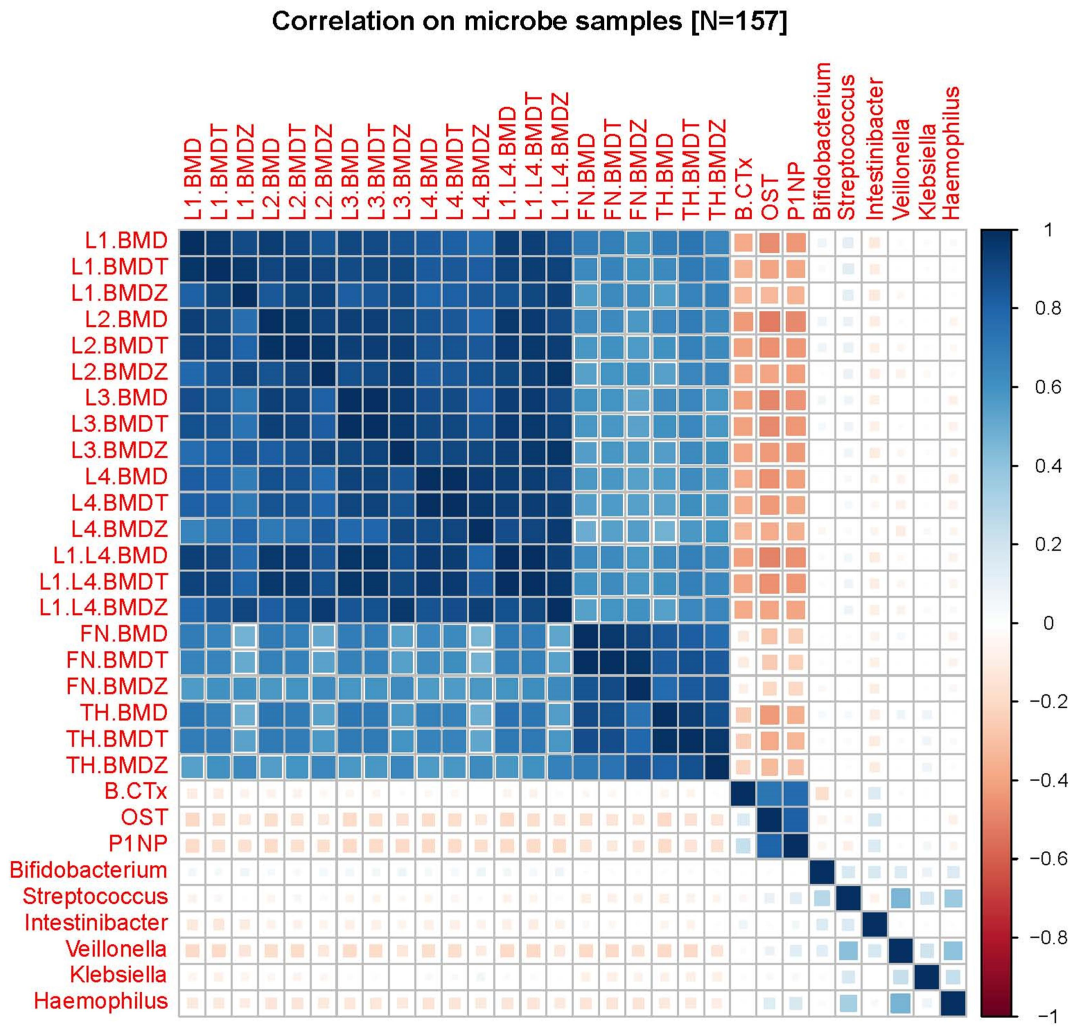

Suppl. Figure 16 - Batch effect and remove of methylation data for CCCO and Jinshan cohorts.

The batch effect is observed in methylation data for CCCO and Jinshan cohorts, where the batch effects remove is effective.

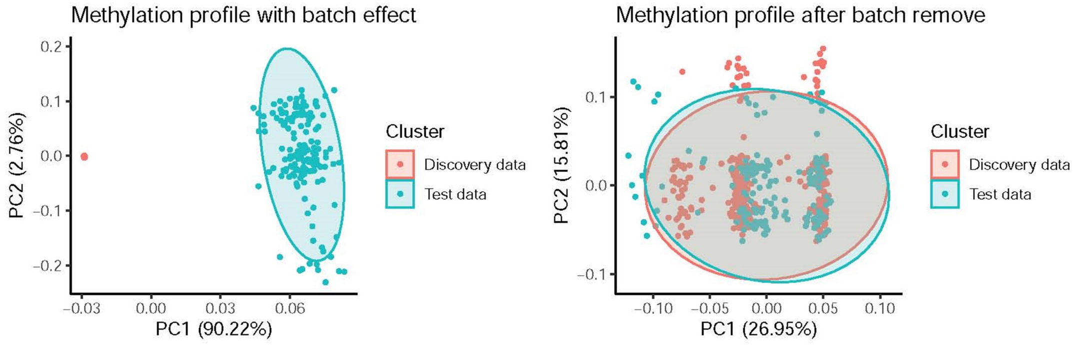

Suppl. Figure 17 - Batch effect and remove of metabolite data for CCCO and Jinshan cohorts.

The batch effect is observed in metabolite data for CCCO and Jinshan cohorts, where the batch effects remove is effective.

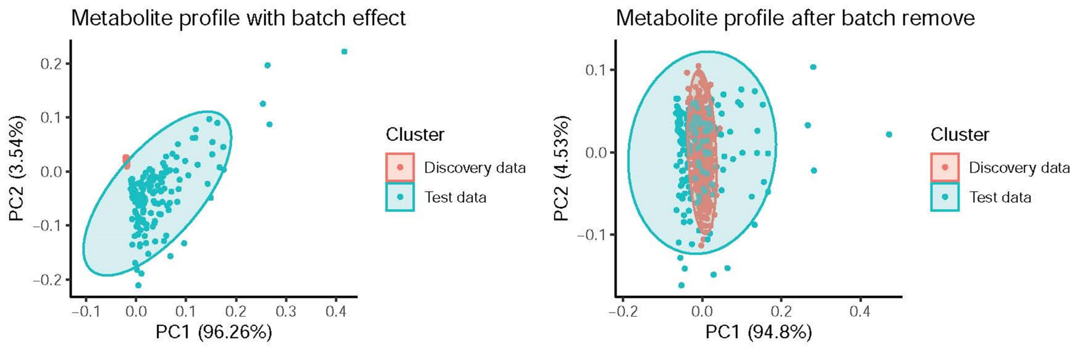

Suppl. Figure 18 - Batch effect and remove of microbiota data for CCCO and Jinshan cohorts.

The batch effect is observed in microbiota data for CCCO and Jinshan cohorts, where the batch effects remove is effective.

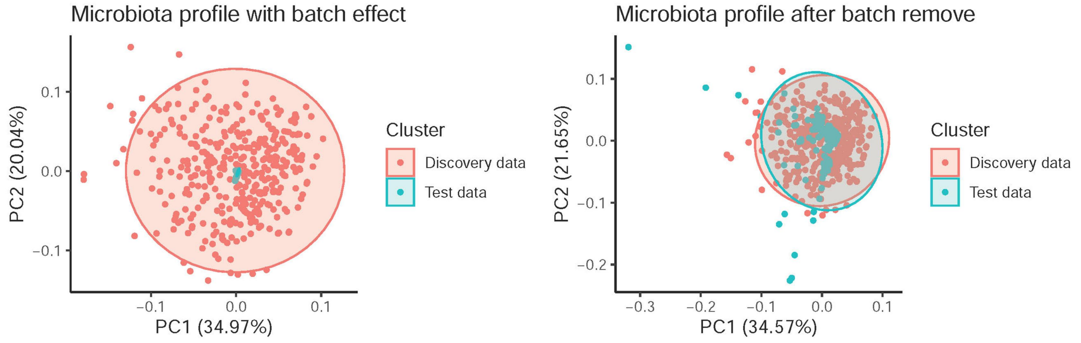

Suppl. Figure 19 - Batch effect of BTMs data for CCCO and Jinshan cohorts.

The batch effect is weak in BTMs data for CCCO and Jinshan cohorts.

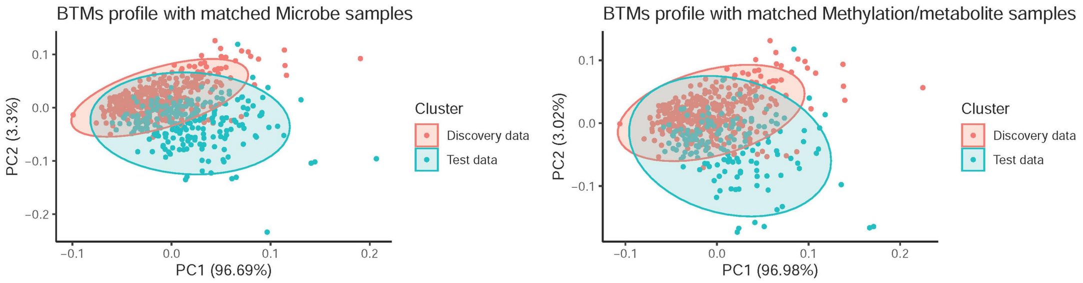

Supp. Figure 20 - Risk model component evaluation for osteoporosis based on metabolite signatures.

As a baseline assessment, risk model for osteoporosis is also built only on the metabolite signatures in M3S.

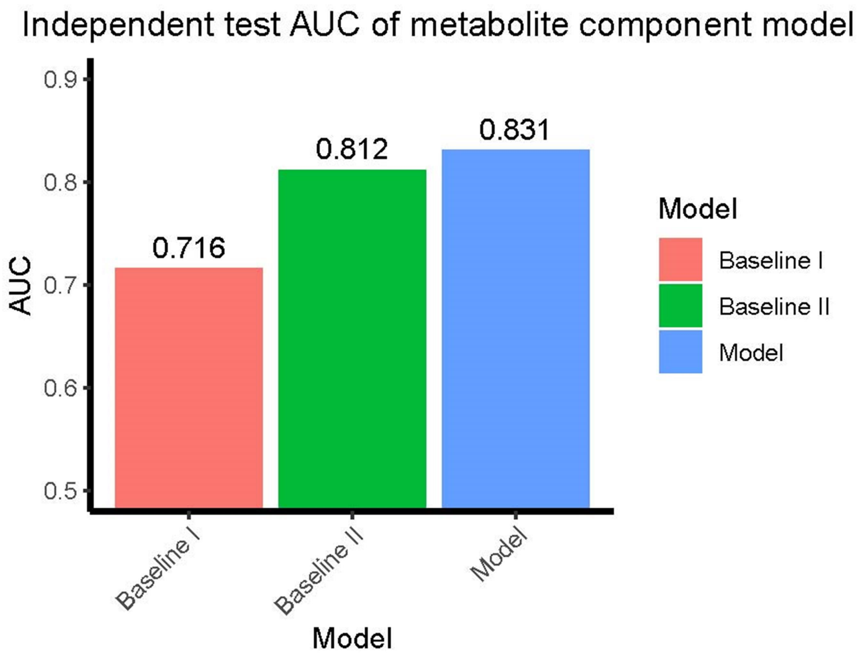

Suppl. Figure 21 - Risk model component evaluation for osteoporosis based on microbiota signatures.

As a baseline assessment, risk model for osteoporosis is also built only on the microbiota signatures in M3S.

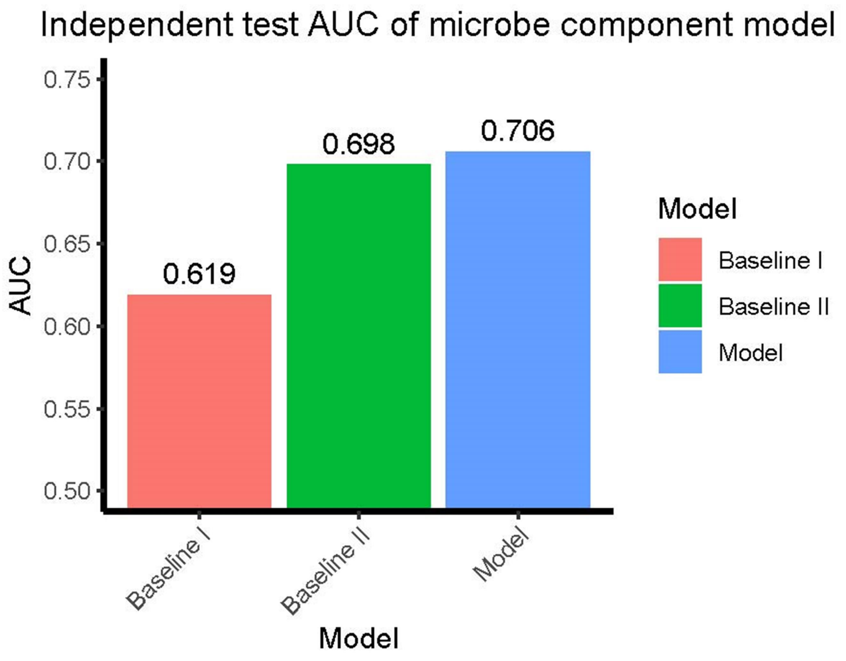

Suppl. Figure 22 - Risk model evaluation for osteoporosis subtypes by prediction associated clinical phenotypes.

The osteoporosis subtype risk model is learned in the CCCO cohort and predicts individuals from the Jinshan cohort, with the predicted two subtypes/groups of Jinshan individuals actually showing significant differences on several key clinical indices.

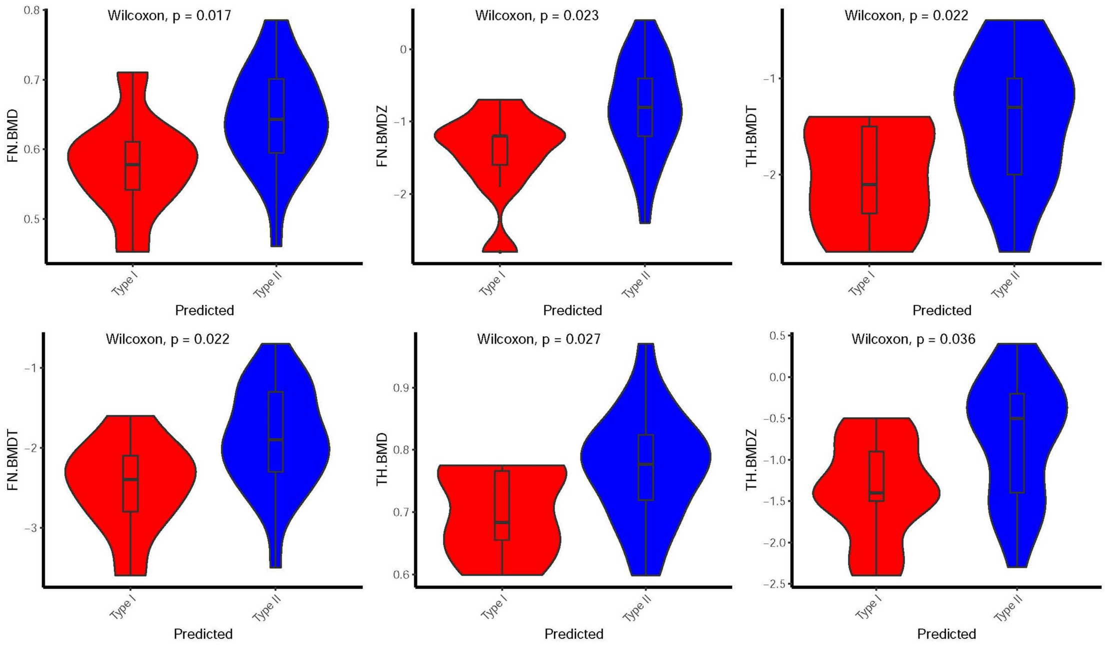

Suppl. Figure 23 - Risk model evaluation with gender as covariate.

a. Decision curve of different risk models with gender as additional covariate (osteoporosis *vs*. others), Baseline I: conventional risk factors; Baseline II: Baseline I + BTMs; Model: Baseline II + our multi-modal molecular signatures (M3S). **b&c**. Independent performance evaluation of risk model (osteoporosis *vs*. others) with gender as additional covariate based on M3S, compared to two baseline methods. **d&e**. Independent performance evaluation of risk model (Yang *vs*. Yin subtypes) with gender as additional covariate based on M3S, compared to other component models and baseline methods. The osteoporosis subtype risk model is learned in the CCCO cohort and predicts individuals from the Jinshan cohort, with the predicted two subtypes/groups of Jinshan individuals showing significant differences on a few clinical indices.

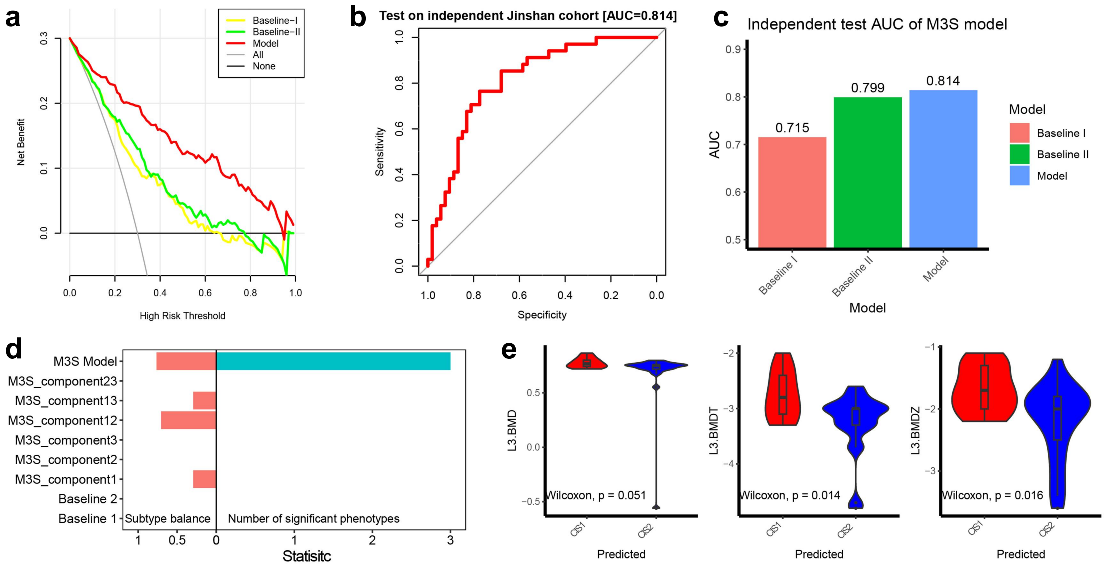

Suppl. Figure 24 – Additional summary of fracture events.

a. Survey of prior fracture (PF) of osteoporosis subtypes. In general, individuals with PF have a higher ratio of (new) fractures. Compared with CIS1, CIS2 individuals tend to have a larger fracture ratio regardless of PF, indicating the higher fracture risk of CIS2. **b**. In addition to age, BMD and PF, there are many molecular factors (e.g. CpGs, metabolites, or microbiotas) associated with fracture events (surveyed in 2023 year) with AUC > 0.6. The association estimated based on original values suggest that many molecular factors would be shared for all population (ALL) or osteoporosis population (OP). After various adjustments, many more osteoporosis specific fracture risk factors would be recognized, which should be independent of BMD. Adjustment I: adjusted with age + gender. Adjustment II: adjusted with age + gender + prior fracture. Adjustment III: adjusted with age + gender + prior fracture + FN BMD.

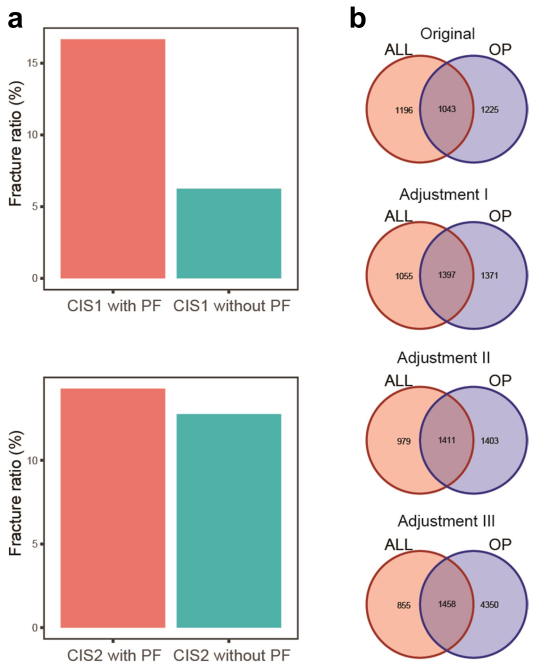

Supplement: Supplementary file 1 — Supplementary Figures 1-24 [file 41421_2024_652_MOESM1_ESM.pdf]
